# Thermodynamic and Kinetic Response of Microbial Reactions to High CO_2_

**DOI:** 10.3389/fmicb.2016.01696

**Published:** 2016-11-17

**Authors:** Qusheng Jin, Matthew F. Kirk

**Affiliations:** ^1^Department of Earth Sciences, University of OregonEugene, OR, USA; ^2^Department of Geology, Kansas State UniversityManhattan, KS, USA

**Keywords:** biogeochemical modeling, available energy, microbial kinetics, carbon sequestration, iron reduction, sulfate reduction

## Abstract

Geological carbon sequestration captures CO_2_ from industrial sources and stores the CO_2_ in subsurface reservoirs, a viable strategy for mitigating global climate change. In assessing the environmental impact of the strategy, a key question is how microbial reactions respond to the elevated CO_2_ concentration. This study uses biogeochemical modeling to explore the influence of CO_2_ on the thermodynamics and kinetics of common microbial reactions in subsurface environments, including syntrophic oxidation, iron reduction, sulfate reduction, and methanogenesis. The results show that increasing CO_2_ levels decreases groundwater pH and modulates chemical speciation of weak acids in groundwater, which in turn affect microbial reactions in different ways and to different extents. Specifically, a thermodynamic analysis shows that increasing CO_2_ partial pressure lowers the energy available from syntrophic oxidation and acetoclastic methanogenesis, but raises the available energy of microbial iron reduction, hydrogenotrophic sulfate reduction and methanogenesis. Kinetic modeling suggests that high CO_2_ has the potential of inhibiting microbial sulfate reduction while promoting iron reduction. These results are consistent with the observations of previous laboratory and field studies, and highlight the complexity in microbiological responses to elevated CO_2_ abundance, and the potential power of biogeochemical modeling in evaluating and quantifying these responses.

## Introduction

Geological carbon sequestration is a potential strategy for stabilizing atmospheric CO_2_ levels despite future increases in fossil fuel combustion (IPCC, 2005). This strategy involves carbon capture—capturing CO_2_ before its emission into the atmosphere—and geological storage—injecting the CO_2_ into subsurface reservoirs (Benson and Cole, 2008). The anticipated depth of storage is >800 m, where CO_2_ can exist in the supercritical state (IPCC, 2005).

Geological carbon storage may negatively affect groundwater resources. CO_2_ or CO_2_-rich brine from storage reservoirs can diffuse through overlying caprocks, and migrate into aquifers via faults, fractures, and abandoned wells (Keating et al., 2013, 2014). The migration of CO_2_ into aquifers can lower groundwater pH, increase salinity, dissolve aquifer minerals, and mobilize hazardous solutes, deteriorating the quality of groundwater (Harvey et al., 2013; Humez et al., 2014; Lions et al., 2014; Shao et al., 2015).

Adding CO_2_ to aquifers may also adversely affect microorganisms. At pressures of 5–35 MPa, CO_2_ can interfere with cell metabolism through a variety of mechanisms, including cytoplasm acidification, membrane lysis, enzyme deactivation, and mobilization of toxic trace elements from mineral surfaces (Watanabe et al., 2003; Bertoloni et al., 2006; Oulé et al., 2006; Wimmer and Zarevúcka, 2010; Santillan et al., 2013). But microbes likely persist in aquifers exposed to high CO_2_ (Kirk et al., 2016a). Many microbes tolerate high CO_2_, especially those that possess Gram-positive cell walls, grow within biofilms, and form spores (Zhang et al., 2006; Mitchell et al., 2008). In addition, microbes survive better in aquifers capable of rapid pH buffering (Wu et al., 2010). Numerous studies have documented microbes in environments of elevated CO_2_, even microbial growth in the presence of supercritical CO_2_ (Yakimov et al., 2002; Inagaki et al., 2006; Videmsek et al., 2009; Oppermann et al., 2010; Emerson et al., 2016; Peet et al., 2015).

However, many questions remain to be addressed. For example, how does CO_2_ affect the thermodynamics and kinetics of microbial reactions in aquifers? How do CO_2_ levels control the outcome of the interactions between different microorganisms? Filling these knowledge gaps is important because microorganisms can affect not only the chemical composition of aquifers but also the flow of groundwater (Gerlach and Cunningham, 2010; Flynn et al., 2013). In addition, many microbial reactions also affect the fate of CO_2_ in aquifers. These reactions consume protons, and hence promote the dissolution and trapping of CO_2_ gas.

In this study, we use biogeochemical modeling to explore how high CO_2_ influences microbial reactions and their interactions in aquifers. Our modeling is based on a simple model that mixes CO_2_ gas into aquifers. Fluxes of CO_2_ gas into aquifers are a direct result of the CO_2_ leakage from deep storage (Carroll et al., 2009). During its migration from storage reservoirs to shallow aquifers, total fluid pressure decreases and supercritical CO_2_ transforms into the phase of gas.

The mixing of CO_2_ gas into groundwater raises the partial pressure of CO_2_. We first explore how the increases in CO_2_ partial pressure impact groundwater chemical properties that are relevant to microbial reactions. We then simulate how the CO_2_ increase affects the thermodynamics of syntrophic oxidation, iron reduction, sulfate reduction, and methanogenesis, common microbial reactions in the subsurface (Lovley and Chapelle, 1995; Bethke et al., 2011). We also carry out kinetic modeling to test the influence of CO_2_ on the activities and interactions of aquifer microorganisms. We then compare the modeling results to the observations of previous laboratory and field investigations. The results show that CO_2_ significantly impacts the thermodynamics and kinetics of microbial reactions, and can change the outcome of microbial interactions.

## Methods

### Hypothetical aquifers

**Figure 1:**
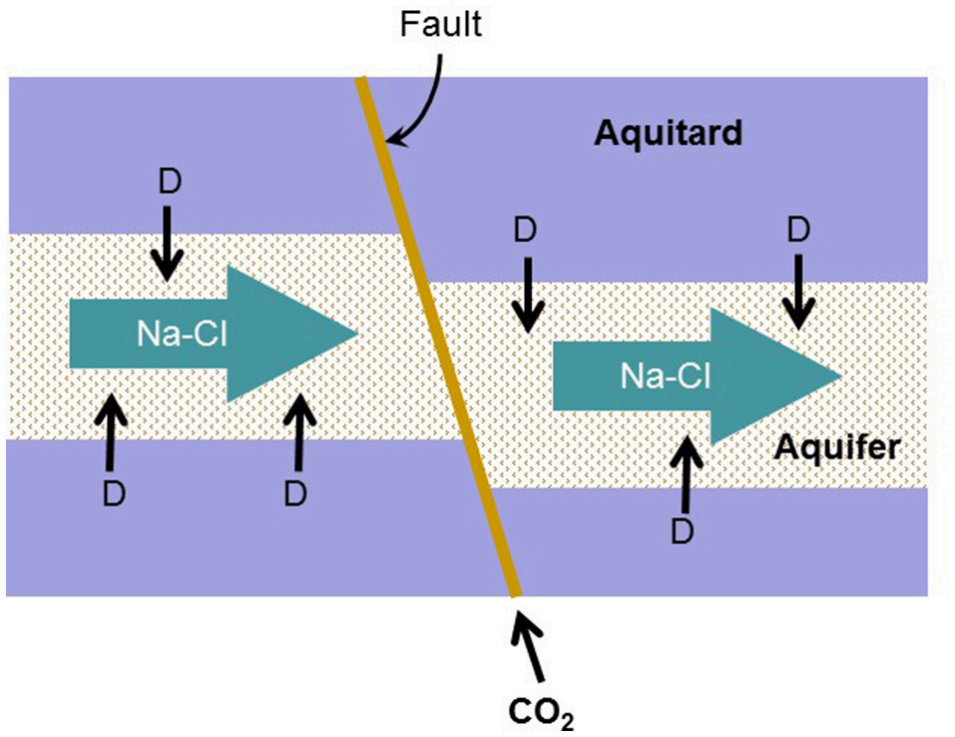
**Conceptual model for biogeochemical reaction modeling**. Na-Cl-Ca water containing sulfate flows through a quartzite aquifer confined between aquitards. In the aquitards, natural organic matter is degraded to H_2_ and acetate (D), which diffuse into the aquifer. CO_2_ from a deep storage reservoir migrates upwards along a fault into the aquifer.

The simulation considers the addition of CO_2_ gas into two hypothetical aquifers, a carbonate-free aquifer and a calcite-rich aquifer (Figure 1). The carbonate-free aquifer has no carbonate mineral, and the calcite-rich aquifer contains abundant calcite as a representative carbonate mineral.

CO_2_ addition into aquifers raises the partial pressure of CO_2_. A wide range of CO_2_ partial pressures are possible, depending on hydrogeological settings and the flux of CO_2_ (Carroll et al., 2009). The simulation considers the partial pressure from near 0 to 30 atm, the range commonly encountered during groundwater sampling. For example, 95% of the wells in the principal aquifers of the United States analyzed by Kirk et al. (2016b) have depths less than 300 m. At this depth, CO_2_ can build up to a maximum partial pressure of 30 atm.

The inclusion of the two aquifers is to account for the wide range in the pH buffering of aquifer minerals. Specifically, in groundwater of circumneutral pH, the addition of CO_2_ induces a hydrolysis reaction:

(1) $$\begin{array}{l} \text{C}{\text{O}}_{2}\left(\text{g}\right)+\;{\text{H}}_{\text{2}}\text{O }⇄\text{C}{\text{O}}_{2}\left(\text{aq}\right)+\;{\text{H}}_{\text{2}}\text{O}⇄{\text{H}}^{+}+\;{\text{HCO}}_{3}^{-}, \end{array}$$

and decreases groundwater pH. The actual pH decrease depends in part on mineral compositions of aquifers (Gunter et al., 1997; Kampman et al., 2009). For example, carbonate minerals can buffer the change in pH by reacting with protons. A common example is the dissolution of calcite (CaCO_3_):

(2) $$\begin{array}{l} \text{Calcite}+\;{\text{H}}^{+}⇄\text{C}{\text{a}}^{2+}+\;{\text{HCO}}_{3}^{-}, \end{array}$$

Which consumes proton and releases calcium and bicarbonate into groundwater.

Other minerals may also contribute to the buffering of pH. For example, the weathering of silicate minerals, such as feldspars and clay minerals, consumes protons, releasing aluminum and silica into groundwater. These reactions are typically much slower than the dissolution of carbonate minerals and hence not as effective as the dissolution of carbonate minerals in pH buffering (Wilkin and Digiulio, 2010). The sorption of protons onto the surface of clay minerals, metal oxides and hydroxides, and other minerals with large surface areas also consumes protons. Compared to mineral dissolution, proton sorption is relatively insignificant in pH buffering (Lions et al., 2014).

### Microbial reactions

Aquifer microbes can be separated into a series of functional groups, including fermenters, syntrophs, and respirers (Jin and Roden, 2011). Fermenting microbes degrade natural organic matter to a series of products, including H_2_, short-chain fatty acids (e.g., acetate, lactate, and propionate), and primary alcohols (e.g., methanol and ethanol). Syntrophs oxidize short-chain fatty acids and primary alcohols to acetate and CO_2_, and transfer the electrons to the reduction of protons to dihydrogen (H_2_). On the other hand, respirers oxidize the products of organic matter degradation, and transfer the released electrons to the reduction of O_2_, ferric minerals, sulfate, bicarbonate, and other electron acceptors.

The redox reactions catalyzed by syntrophs and respirers can be represented as:

(3) $$\begin{array}{l} \underset{\text{D}}{\sum }\nu_{\text{D}}\text{D}+\underset{\text{A}}{\sum }\nu_{\text{A}}\text{A}\vec{\leftarrow }\underset{{\text{D}}^{+}}{\sum }\nu_{{\text{D}}^{+}}{\text{D}}^{+}+\underset{{\text{A}}^{-}}{\sum }\nu_{{\text{A}}^{-}}{\text{A}}^{-}, \end{array}$$

where D and D^+^ are electron donors and their oxidized forms, respectively, A and A^−^ are electron acceptors and their reduced forms, respectively, and ν_D_ and others are stoichiometric coefficients. In microbiology and biochemistry, the thermodynamics of redox reactions is commonly characterized using reduction potential. Specifically, for the redox couple of D and D^+^, the reduction potential *E*_D_ (V) is calculated according to:

(4) $$\begin{array}{l} E_{\text{D}}=E_{\text{D}}^{\text{o}}\prime -\frac{RT}{nF}\cdot \;\left[\ln\left(\prod_{\text{D}}\gamma_{\text{D}}^{\nu_{\text{D}}}\;\cdot \;m_{\text{D}}^{\nu_{\text{D}}}\right)-\ln\left(\prod_{{\text{D}}^{+}}\gamma_{{\text{D}}^{+}}^{\nu_{{\text{D}}^{+}}}\;\cdot \;m_{{\text{D}}^{+}}^{\nu_{{\text{D}}^{+}}}\right)\right]; \end{array}$$

for the redox couple of A and A^−^, the reduction potential *E*_A_ is calculated as:

(5) $$E_{\text{A}}=E_{\text{A}}^{\text{o}}\prime -\frac{RT}{nF}\cdot \;\left[\ln\left(\prod_{{\text{A}}^{-}}\gamma_{{\text{A}}^{-}}^{\nu_{{\text{A}}^{-}}}\;\cdot \;m_{{\text{A}}^{-}}^{\nu_{{\text{A}}^{-}}}\right)-\ln\left(\prod_{\text{A}}\gamma_{\text{A}}^{\nu_{\text{A}}}\;\cdot \;m_{\text{A}}^{\nu_{\text{A}}}\right)\right].\;$$

Here $E_{\text{D}}^{\text{o}}\prime \;$ and $E_{\text{A}}^{\text{o}}\prime \;$ are standard potentials at pH 7, *n* is the number of electrons transferred per reaction, γ_D_ and others are activity coefficients (M^−1^), *m*_*D*_ and others are molal concentrations, *R* is the gas constant (J · mol^−1^ · K^−1^), *F* is the Faraday's constant, and *T* is the absolute temperature (K). Table 1 lists the reduction reactions of redox couples commonly found in aquifers and the standard reduction potentials ($E_{\text{D}}^{\text{o}}\prime \;$ and $E_{\text{A}}^{\text{o}}\prime \;$) at 1 atm, 25°C, and pH 7. For the purpose of comparing stoichiometric coefficients of proton and bicarbonate, the reactions are written in terms of 8 electrons (*n* = 8).

**Table 1:** **Reduction reactions of common electron donors and acceptors in aquifers, and their standard reduction potentials ***E***^o′^ at pH 7^a^**.

**Half-reaction**	***E*^o′^ (mV)**
$\text{8}{\text{H}}^{+}+8{\text{e}}^{-}\to 4{\text{H}}_{2}\left(\text{aq}\right)$	−506.2
2Acetate + $2{\text{HCO}}_{3}^{-}+{\text{10H}}^{+}+8{\text{e}}^{-}\to 2\text{Lactate}+4{\text{H}}_{2}\text{O}$	−438.0
2Acetate +10${\text{H}}^{+}+8{\text{e}}^{-}\to 2\text{Ethanol}+2{\text{H}}_{2}\text{O}$	−390.3
8Goethite $+\;\text{24}{\text{H}}^{+}+8{\text{e}}^{-}\to 8\text{F}{\text{e}}^{2+}+\text{16}{\text{H}}_{2}\text{O}$	−389.7
$\frac{4}{3}{\text{HCO}}_{3}^{-}+\frac{28}{3}{\text{H}}^{+}+8{\text{e}}^{-}\to \frac{4}{3}\text{Methanol}+\frac{8}{3}{\text{H}}_{2}\text{O}$	−373.7
$\text{4Acetate}+10{\text{H}}^{+}+8{\text{e}}^{-}\to 2\text{Butyrate}+4{\text{H}}_{2}\text{O}$	−284.8
${\text{2HCO}}_{3}^{-}+9{\text{H}}^{+}+8{\text{e}}^{-}\to \text{Acetate}+4{\text{H}}_{2}\text{O}$	−279.1
$\frac{4}{3}\text{Acetate}+\frac{4}{3}{\text{HCO}}_{3}^{-}+\frac{28}{3}{\text{H}}^{+}+8{\text{e}}^{-}\to \frac{4}{3}\text{Propionate}+4{\text{H}}_{2}\text{O}$	−278.7
${\text{HCO}}_{3}^{-}+9{\text{H}}^{+}+8{\text{e}}^{-}\to \text{C}{\text{H}}_{4}\left(\text{aq}\right)+3{\text{H}}_{2}\text{O}$	−259.6
${\text{SO}}_{4}^{2-}+10{\text{H}}^{+}+8{\text{e}}^{-}\to {\text{H}}_{2}\text{S}+4{\text{H}}_{2}\text{O}$	−165.6

a *Standard reduction potential at 1 atm, 25°C, and pH 7 is calculated from the updated LLNL Thermodynamic Database (Delany and Lundeen, 1990)*.

By transferring electrons, syntrophs and respirers liberate the chemical energy from redox reactions, which becomes available to their metabolisms. The available energy Δ*G*_A_ [J · (mol reaction)^−1^, or J · mol^−1^] is the negative of the Gibbs free energy change of redox reactions, and is calculated from:

(6) $$\Delta G_{\text{A}}=nF\;\cdot \;\left(E_{\text{A}}-E_{\text{D}}\right),$$

the difference in the reduction potentials between electron acceptors *E*_A_ and donors *E*_D_. Table 2 lists the standard available energy at 1 atm, 25°C, and pH 7 for common redox reactions in aquifers. Following previous practice (Bethke et al., 2011; Jin, 2012), we compute the energy available from microbial reactions that transfer 8 electrons, or the consumption of one acetate or four dihydrogen molecules (Table 2).

**Table 2:** **Redox reactions, and their standard available energy $\Delta G_{\text{A}}^{o}\prime$ at pH 7, 1 atm and 25°C**.

**Redox reaction**	**$\Delta G_{\text{A}}^{o}\prime$ (kJ.mol^−1^)**
**SYNTROPHIC OXIDATION**	
1. $\text{Acetate}\;+\;4{\text{H}}_{2}\text{O}\;\vec{\leftarrow }\;4{\text{H}}_{2}\left(\text{aq}\right)\;+\;{\text{2HCO}}_{3}^{-}\;+\;{\text{H}}^{+\;}$	−175.25
2. $\text{2Lactate}\;+\;4{\text{H}}_{2}\text{O}\;\vec{\leftarrow }\;2\text{Acetate}\;+\;4{\text{H}}_{2}\left(\text{aq}\right)\;+\;{\text{2HCO}}_{3}^{-}\;+\;\text{2}{\text{H}}^{+\;}$	−52.65
3. $\frac{4}{3}\text{Propionate}\;+\;4{\text{H}}_{2}\text{O}\;\vec{\leftarrow }\;\frac{4}{3}\text{Acetate}\;+\;4{\text{H}}_{2}\left(\text{aq}\right)\;+\;\frac{4}{3}{\text{HCO}}_{3}^{-}\;+\;\frac{4}{3}{\text{H}}^{+\;}$	−175.58
4. $\text{2Butyrate}\;+\;4{\text{H}}_{2}\text{O}\;\vec{\leftarrow }\;\text{4Acetate}\;+\;4{\text{H}}_{2}\left(\text{aq}\right)\;+\;2{\text{H}}^{+\;}$	−170.90
5. $\frac{4}{3}\text{Methanol}\;+\;\frac{8}{3}{\text{H}}_{2}\text{O}\;\vec{\leftarrow }\;4{\text{H}}_{2}\left(\text{aq}\right)\;+\;\frac{4}{3}{\text{HCO}}_{3}^{-}\;+\;\frac{4}{3}{\text{H}}^{+\;}$	−102.24
6. $\text{2Ethanol}\;+\;2{\text{H}}_{2}\text{O}\;\vec{\leftarrow }\;2\text{Acetate}\;+\;4{\text{H}}_{2}\left(\text{aq}\right)\;+\;\text{2}{\text{H}}^{+\;}$	−89.42
**GOETHITE REDUCTION**	
7. $\text{4}{\text{H}}_{2}\left(\text{aq}\right)\;+\;\text{8Goethite}\;+\;16{\text{H}}^{+\;}\;\vec{\leftarrow }\;\text{16}{\text{H}}_{2}\text{O}\;+\;8\text{F}{\text{e}}^{2+\;}$	89.90
8. $\text{Acetate}\;+\;\text{8Goethite}\;+\;15{\text{H}}^{+\;}\;\vec{\leftarrow }\;{\text{2HCO}}_{3}^{-}\;+\;12{\text{H}}_{2}\text{O}\;+\;8\text{F}{\text{e}}^{2+\;}$	−85.35
9. $\text{2Lactate}\;+\;\text{8Goethite}\;+\;14{\text{H}}^{+\;}\;\vec{\leftarrow }\;2\text{Acetate}\;+\;{\text{2HCO}}_{3}^{-}\;+\;12{\text{H}}_{2}\text{O}\;+\;8\text{F}{\text{e}}^{2+\;}$	37.25
10. $\frac{4}{3}\text{Propionate}\;+\;\text{8Goethite}\;+\;12\frac{2}{3}{\text{H}}^{+\;}\;\vec{\leftarrow }\;\frac{4}{3}\text{Acetate}\;+\;\frac{4}{3}{\text{HCO}}_{3}^{-}\;+\;12{\text{H}}_{2}\text{O}\;+\;8\text{F}{\text{e}}^{2+\;}$	−85.68
11. $2\text{Butyrate}\;+\;\text{8Goethite}\;+\;14{\text{H}}^{+\;}\;\vec{\leftarrow }\;\text{4Acetate}\;+\;12{\text{H}}_{2}\text{O}\;+\;8\text{F}{\text{e}}^{2+\;}$	−81.00
12. $\frac{4}{3}\text{Methanol}\;+\;\text{8Goethite}\;+\;\frac{\text{44}}{3}{\text{H}}^{+\;}\;\vec{\leftarrow }\;\frac{4}{3}{\text{HCO}}_{3}^{-}\;+\;8\text{F}{\text{e}}^{2+\;}\;+\;\frac{40}{3}{\text{H}}_{2}\text{O}$	−12.34
13. $2\text{Ethanol}\;+\;\text{8Goethite}\;+\;14{\text{H}}^{+\;}\;\vec{\leftarrow }\;\text{2Acetate}\;+\;8\text{F}{\text{e}}^{2+\;}\;+\;14{\text{H}}_{2}\text{O}$	0.48
**SULFATE REDUCTION**	
14. $4{\text{H}}_{2}\text{(aq)}\;+\;{\text{SO}}_{4}^{2-}\;+\;2{\text{H}}^{+\;}\;\vec{\leftarrow }\;{\text{H}}_{2}\text{S}\;+\;4{\text{H}}_{2}\text{O}$	223.23
15. $\text{Acetate}\;+\;{\text{SO}}_{4}^{2-}\;+\;{\text{H}}^{+\;}\;\vec{\leftarrow }\;{\text{2HCO}}_{3}^{-}\;+\;{\text{H}}_{2}\text{S}$	47.97
16. $\text{2Lactate}\;+\;{\text{SO}}_{4}^{2-}\;\vec{\leftarrow }\;2{\text{Acetate + 2HCO}}_{3}^{-}\;+\;{\text{H}}_{2}\text{S}$	170.57
17. $\frac{4}{3}\text{Propionate}\;+\;{\text{SO}}_{4}^{2-}\;+\;\frac{2}{3}{\text{H}}^{+\;}\;\vec{\leftarrow }\;\frac{4}{3}\text{Acetate}\;+\;\frac{4}{3}{\text{HCO}}_{3}^{-}\;+\;{\text{H}}_{2}\text{S}$	47.64
18. $2\text{Butyrate}\;+\text{ S}{\text{O}}_{4}^{2-}\;\vec{\leftarrow }\;\text{4Acetate}\;+\;{\text{H}}_{2}\text{S}$	52.33
19. $\frac{4}{3}\text{Methanol}\;+\;{\text{SO}}_{4}^{2-}\;+\;\frac{2}{3}{\text{H}}^{+\;}\;\vec{\leftarrow }\;{\text{H}}_{2}\text{S}\;+\;\frac{\text{4}}{3}{\text{H}}_{2}\text{O}\;+\;\frac{4}{3}{\text{HCO}}_{3}^{-}$	120.98
20. $2\text{Ethanol}\;+\;{\text{SO}}_{4}^{2-}\;\vec{\leftarrow }\;\text{2Acetate}\;+\;{\text{H}}_{2}\text{S}\;+\;2{\text{H}}_{2}\text{O}$	133.80
**METHANOGENESIS**	
21. $\text{4}{\text{H}}_{2}\text{(aq)}\;+\;{\text{H}}^{+\;}\;+\;{\text{HCO}}_{3}^{-}\;\vec{\leftarrow }\;\text{C}{\text{H}}_{4}\left(\text{aq}\right)\;+\;3{\text{H}}_{2}\text{O}$	190.33
22. $\text{Acetate}\;+\;{\text{H}}_{2}\text{O}\;\vec{\leftarrow }\;{\text{HCO}}_{3}^{-}\;+\;\text{C}{\text{H}}_{4}\left(\text{aq}\right)$	15.07

The rate *r* (mol · L^−1^ · s^−1^) of microbially-driven redox reactions can be calculated according to the thermodynamically consistent rate law (Jin and Bethke, 2003, 2005, 2007):

(7) $$\begin{array}{l} r=k\cdot \left[\text{X}\right]\cdot F_{\text{D}}\cdot F_{\text{A}}\cdot F_{\text{T}}, \end{array}$$

where *k* is the rate constant [mol · (g dry weight)^−1^ · s^−1^, or mol · g^−1^ · s^−1^], [X] is the biomass concentration [g dry weight · L^−1^, or g · L^−1^], *F*_D_ and *F*_A_ are the kinetic factors of electron donor and acceptor, respectively, and *F*_T_ is the thermodynamic potential factor. The kinetic factors are calculated according to:

(8) $$\begin{array}{l} F_{\text{D}}=\frac{m_{\text{D}}}{K_{\text{D}}+m_{\text{D}}}, \end{array}$$

and

(9) $$\begin{array}{l} F_{\text{A}}=\frac{m_{\text{A}}}{K_{\text{A}}+m_{\text{A}}}, \end{array}$$

where *K*_D_ and *K*_A_ are the half-saturation constants (M) for electron donor D and acceptor A, respectively. The thermodynamic factor is calculated according to:

(10) $$\begin{array}{l} F_{\text{T}}=1-\exp\left(-\frac{\Delta G_{\text{A}}-\Delta G_{\text{C}}}{\chi \cdot RT}\right) \end{array}$$

where Δ*G*_C_ (J · mol^−1^) represents the energy saved by microbes, and χ is the average stoichiometric number. The saved energy Δ*G*_C_ is calculated as:

(11) $$\begin{array}{l} \Delta G_{\text{C}}=m_{\text{P}}\cdot \Delta G_{\text{P}}, \end{array}$$

the product of the ATP yield *m*_P_ of microbial reaction and the phosphorylation energy Δ*G*_P_. The phosphorylation energy is the energy required to synthesize ATP from ADP and phosphate in the cytoplasm. In this study, its value is taken as 45 kJ · (mol ATP)^−1^ (Jin, 2012).

For microbial reduction of ferric minerals, its rate depends on the molal concentration *m*_surf, avail_ of bioavailable surface sites of the minerals. According to Roden (2006), the rate can be calculated according to:

(12) $$\begin{array}{l} r=k_{\text{surf}}\cdot m_{\text{surf,avail}}\cdot \frac{\left[\text{X}\right]/m_{\text{surf,avail}}}{K_{\text{A}}^{\text{surf,avail}}+\left[\text{X}\right]/m_{\text{surf,avail}}}\cdot F_{\text{D}}\cdot F_{\text{T}}, \end{array}$$

where *k*_surf_ is the bioavailable site-specific rate constant (s^−1^), and $K_{\text{A}}^{\text{surf,avail}}$ is a constant in g cell dry weight per mol bioavailable surface sites (g · mol^−1^).

Syntrophs and respirers utilize the saved energy Δ*G*_C_ to synthesize biomass. The rate at which the biomass concentration [X] changes with time is:

(13) $$\begin{array}{l} \frac{d\left[\text{X}\right]}{dt}=\left(\mu -D\right)\cdot \left[\text{X}\right], \end{array}$$

where μ is the specific growth rate (s^−1^), and *D* is the specific rate of maintenance (s^−1^). The specific growth rate μ is calculated according to:

(14) $$\begin{array}{l} \mu =Y\cdot \frac{r}{\left[\text{X}\right]}. \end{array}$$

Here *Y* is the growth yield, the grams of biomass synthesized per mol reaction (g · mol^−1^).

### Model implementation

We carried out the simulation using the React program of the software package Geochemist's Workbench version 9.0 (Bethke, 2008). The simulation assumes that aqueous chemical speciation, mineral dissolution and precipitation, and ion sorption are at thermodynamic equilibrium, and describes these reactions on the basis of the updated LLNL Thermodynamic Database (Delany and Lundeen, 1990). This database was modified to include amorphous iron sulfide (solubility product of 10^−2.96^) (Langmuir, 1997), and goethite (solubility product of 10^1.40^) (Bigham et al., 1996). The activity coefficients are calculated according to an extended form of the Debye-Hückel equation (Helgeson, 1969).

The simulation describes the sorption of ferrous iron onto the surface of goethite using non-electrostatic Langmuir isotherm (Stumm and Morgan, 1996). Specifically, the sorption reaction is:

(15) $$\begin{array}{l} >\text{FeOH}+\text{F}{\text{e}}^{2+}⇄>\text{FeOF}{\text{e}}^{+}+{\text{H}}^{+}, \end{array}$$

where >FeOH represents the native surface site available to bioreduction, and >FeOFe^+^ is ferrous iron surface species. The logarithmic equilibrium constant of the reaction is −2.5 (Appelo et al., 2002). The concentrations of the surface sites are calculated by taking a standard site density of 3.84 × 10^−6^ mol × m^−2^ (Davis and Kent, 1990) and a specific surface area of 225 m^2^ × g^−1^ for goethite (Roden, 2004). The concentration *m*_surf, avail_ of bioavailable surface site is calculated as the difference in concentration between the total surface sites and those occupied by ferrous iron.

## Results and discussion

### Groundwater chemistry

The simulation assumes that both the hypothetical carbonate-free and calcite-rich aquifers contain the same Na-Cl-Ca type groundwater. According to Kirk et al. (2016b), in 18 principal aquifer systems of the United States, Ca^2+^ has an average concentration of 1.4 mM, and four aquifer systems have average pH values near 8.0. Thus, the simulation assumes that groundwater in the two hypothetical aquifers has pH of 8 and contains 10 mM Na^+^, 10 mM Cl^−^, and 2.0 mM Ca^2+^.

**Figure 2:**
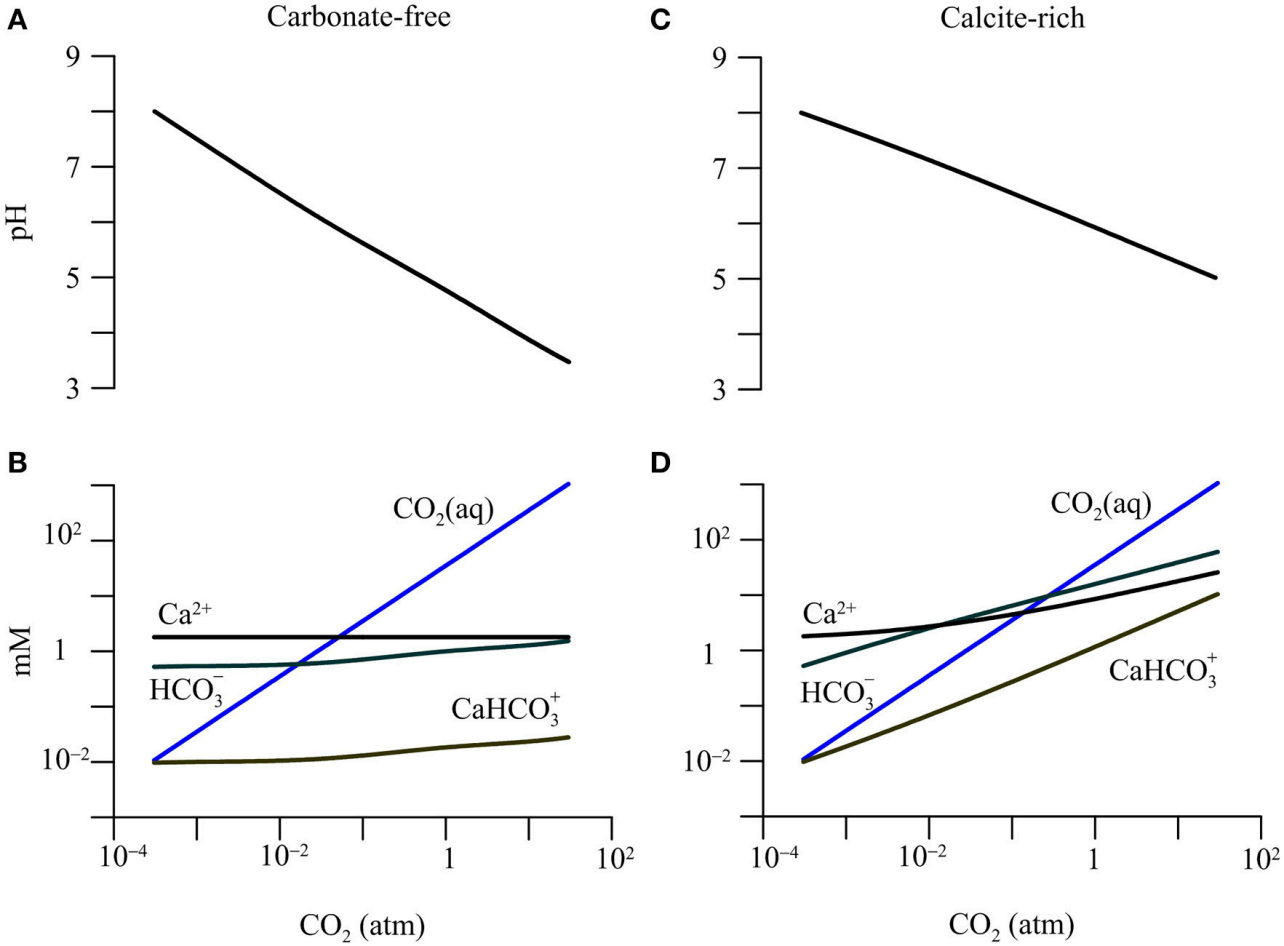
**Variations in pH and concentrations of calcium (Ca^**2+**^), dissolved CO_**2**_(aq), bicarbonate (${\text{HCO}}_{3}^{-}$), and calcium-bicarbonate complex (${\text{CaHCO}}_{3}^{+}$) with CO_**2**_ partial pressure in a hypothetical carbonate-free (A,B)** and calcite-rich aquifer **(C,D)**.

**Figure 3:**
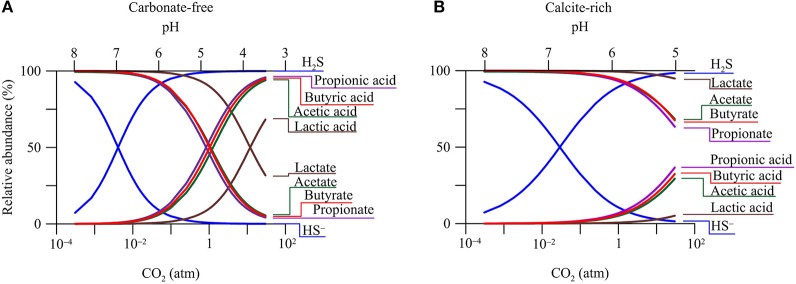
**Variations in relative abundances of monohydrogen sulfide (HS^**−**^), acetate, lactate, propionate, butyrate, and their conjugate acids with CO_**2**_ partial pressure in a hypothetical carbonate-free (A)** and calcite-rich aquifer **(B)**.

**Figure 4:**
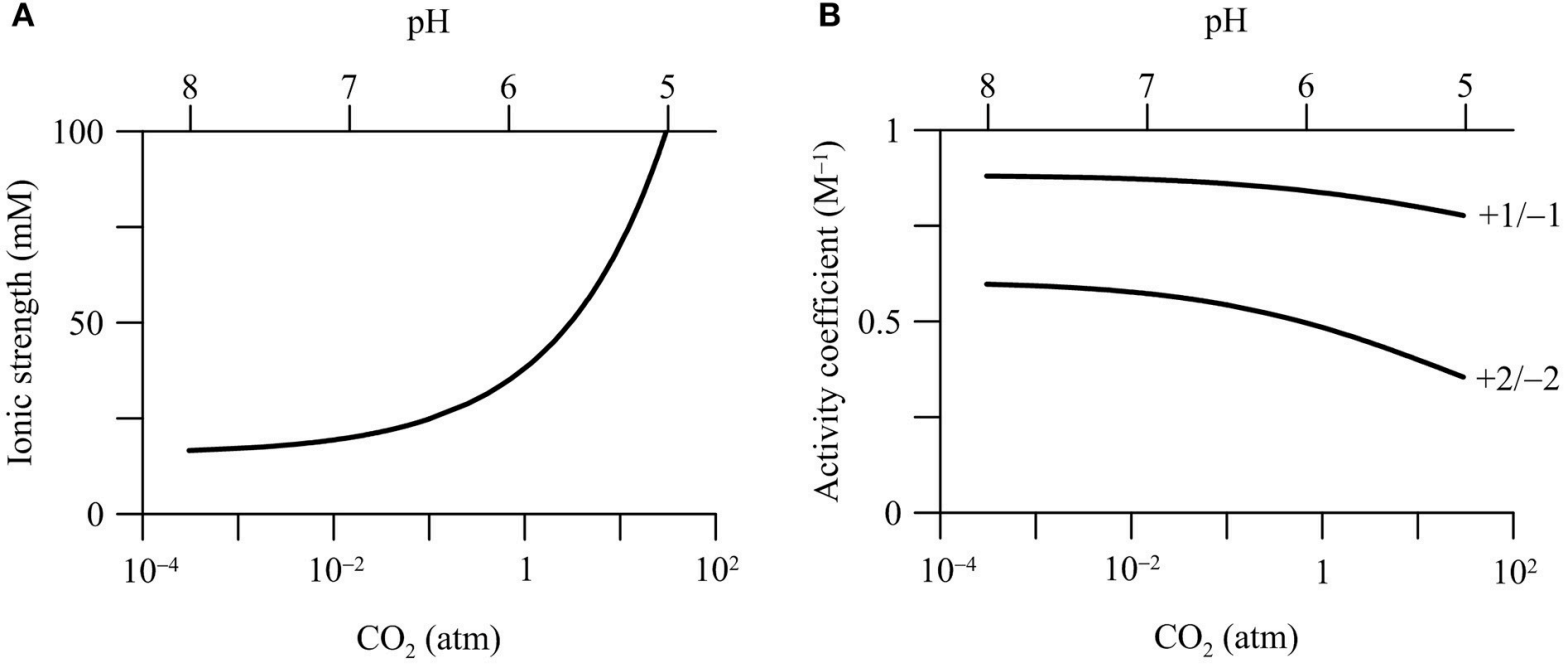
**Variations in the ionic strength of groundwater (A)** and the activity coefficients of ions **(B)** with CO_2_ partial pressure in a hypothetical calcite-rich aquifer. Labels show the charges of ions.

The simulation also assumes that in both aquifers, the groundwater is equilibrated with the precipitation or dissolution of calcite. Under this assumption, in both aquifers, the partial pressure of CO_2_ is 3.1 × 10^−4^ atm, and the dissolved inorganic carbon occurs mainly as bicarbonate (0.5 mM), dissolved CO_2_ (0.01 mM), and a calcium-bicarbonate complex species (0.01 mM). Figures 2–**4** show, according to the simulation results, how the groundwater chemistry responds to the addition of CO_2_ gas into the aquifers.

#### pH and inorganic carbon

The addition of CO_2_ lowers groundwater pH and raises the concentration of dissolved CO_2_ (Figure 2). Specifically, in the carbonate-free aquifer, where the partial pressure increases to 30 atm, pH decreases to 3.5, and dissolved CO_2_ concentration increases to 1.06 M. But there is relatively little increase in the concentration of bicarbonate or the calcium-bicarbonate complex (${\text{CaHCO}}_{3}^{+}$).

In the calcite-rich aquifer, the increase in CO_2_ partial pressure to 30 atm also raises the concentration of dissolved CO_2_ to 1.06 M, but lowers groundwater pH only to 5. In addition, the concentrations of bicarbonate and ${\text{CaHCO}}_{3}^{+}$ increase to 60.5 mM and 10.4 mM, respectively.

The different responses of the two aquifers arise from CO_2_-induced dissolution of calcite. In the carbonate-free aquifer, the simulation does not consider any reaction that consumes protons. As a result, most of the protons generated by CO_2_ addition and hydrolysis largely stay in the groundwater, lowering pH significantly. In comparison, in the calcite-rich aquifer, protons react with calcite, which buffers the decrease in pH, and adds bicarbonate and calcium to the groundwater (Equations 1 and 2).

In addition to pH buffering, the pH decrease by CO_2_ addition also depends on the pressure, temperature, and salinity of subsurface fluids. The solubility of CO_2_ increases with pressure, and thus depth, but decreases with temperature and salinity (Benson and Cole, 2008). Nevertheless, the simulation results agree with previous assessment. For example, several field and laboratory studies reported that CO_2_ addition decreased groundwater pH by 0.8–2.9 units (Lions et al., 2014). Similarly, geochemical modeling analysis indicated that, during the CO_2_ injection experiment in the Frio Formation, the *in situ* pH of groundwater decreased from about 6.5 to 3 (Kharaka et al., 2006).

#### Aqueous speciation

The pH decrease in groundwater has a direct impact on the speciation of dissolved chemicals. Figure 3 shows how CO_2_ changes the relative abundances of acetate, lactate, propionate, butyrate, monohydrogen sulfide (HS^−^), and their conjugate acids in the two hypothetical aquifers.

The response of aqueous speciation is more pronounced in the carbonate-free aquifer than in the calcite-rich aquifer. In the carbonate-free aquifer (Figure 3A), the relative abundances of different acids increase, while those of the conjugate bases decrease, with the increase in CO_2_ partial pressure. The appearance of the cross-over points for the acids and their conjugate bases follows the sequence of acidity constant. Among these acids, dihydrogen sulfide (H_2_S) has the largest logarithmic acidity constant (pKa) of 7.0 (Lide, 2003), and H_2_S and HS^−^ reach equal concentrations where CO_2_ partial pressure increases to 4.0 × 10^−3^ atm, and groundwater pH decreases to 7.0. On the other hand, lactic acid has the smallest pKa of 3.86, and lactic acid and lactate take the same concentration where CO_2_ partial pressure reaches 12 atm, and groundwater pH drops to 3.86.

In the calcite-rich aquifer (Figure 3B), the speciation of dihydrogen sulfide shows significant variations. At CO_2_ partial pressure of 2.9 × 10^−2^ atm and pH of 7, the two species have the same concentration. The speciations of acetic acid, propionic acid, and butyric acid also respond to the increase in CO_2_ partial pressure, but to much lesser extents. These acids occur at notable concentrations, only after CO_2_ partial pressure increases to over 1 atm and pH decreases to 6. These modest responses reflect the limited decrease in groundwater pH (Figure 2C).

#### Ionic strength and activity coefficient

In the calcite-rich aquifer, CO_2_ addition raises the ionic strength of the groundwater (Figure 4A). Where CO_2_ partial pressure increases from 3.1 × 10^−4^ to 30 atm, the ionic strength increases from 16.6 to 100 mM. This increase is mainly due to increases in the concentrations of Ca^2+^ and bicarbonate by the dissolution of calcite (Figure 2D). In comparison, in the carbonate-free aquifer, the ionic strength of groundwater remains nearly constant with the increase in CO_2_ partial pressure (data not shown).

Increases in the ionic strength are commonly observed in previous field investigations. For example, Kharaka et al. (2010), Trautz et al. (2013) and Cahill et al. (2013) all reported temporary increases in the electrical conductivity and the concentrations of bicarbonate, Ca^2+^, and other ions during the injection of CO_2_ into shallow aquifers. Note that in our simulation, the increase of the ionic strength is due to calcite dissolution. Other factors, such as sorption reactions and mixing with deep reservoir fluids, can also raise groundwater ionic strength (Jones et al., 2015).

Ionic strength controls the thermodynamic properties of dissolved chemical species, which can be quantified using activity coefficient (see Equations 4 and 5). Among different approaches, the extended Debye-Hückel equation, or the B-dot equation, represents a robust choice for Na/Cl-dominated groundwater with ionic strength up to 2 molal (Helgeson, 1969). According to the B-dot equation, activity coefficients depend significantly on the ionic strength and the charges of chemical species. Figure 4B shows that the activity coefficients of ions decrease with the increases in CO_2_ partial pressure in the calcite-rich aquifer. For chemical species with +1 or −1 charge, where CO_2_ partial pressure increases from near 0 to 30 atm, the activity coefficients decrease by about 0.1, from near 0.89 to 0.77. For those with +2 or −2 charge, the activity coefficients decrease by about 0.2, from near 0.6 to 0.4. For neutral chemical species, the activity coefficients are set at unity, and do not vary with the ionic strength.

### Reduction potential

The above geochemical variations place a fundamental constraint on the reduction potentials of redox couples in microbial reactions. Here we focus on the electron donors produced by the degradation of natural organic matter, including dihydrogen, acetate, lactate, propionate, butyrate, methanol, and ethanol, and consider the common electron acceptors in aquifers, such as goethite, sulfate, bicarbonate, and proton (Lovley and Chapelle, 1995; Bethke et al., 2011).

We compute the changes in reduction potentials with CO_2_ partial pressures, not their absolute values, in order to highlight the responses of redox couples and to compare the responses among different redox couples. Using the changes, not absolute values, also avoids the need of absolute concentrations of electron donors, acceptors, and reaction products. In aquifers, there are few concentration measurements for lactate, propionate, butyrate, methanol, and ethanol. On the other hand, the concentrations of ferrous iron, sulfate, sulfide, and bicarbonate vary over orders of magnitude (Kirk et al., 2016b). Using the changes also simplifies the discussion of ferric mineral reduction. There are different ferric minerals in aquifers, such as ferrihydrite, goethite, hematite, and lepidocrocite. Their reduction potentials are different (Cornell and Schwertmann, 2003), but respond in the same fashion to pH, because the reduction of these ferric minerals consumes the same number of protons per electron. Here we take goethite as an example, but the results are applicable to ferrihydrite, hematite, and lepidocrocite.

**Figure 5:**
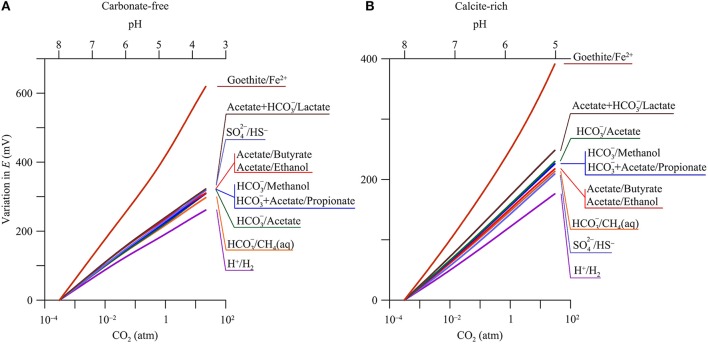
**Variations in reduction potentials ***E*** of redox couples with CO_**2**_ partial pressure in a hypothetical carbonate-free (A)** and calcite-rich aquifer **(B)**. Labels show the redox couples; see Table 1 for reduction reactions.

Figure 5 shows, according to the simulation results, how the reduction potentials respond to CO_2_ partial pressure in both the carbonate-free and calcite-rich aquifers. The reduction potentials increase with CO_2_ partial pressures. For the redox couples considered by this study (see Table 1), their reduction reactions consume protons and, as a result, their reduction potentials increase with the decrease in groundwater pH (Equations 4 and 5).

#### Carbonate-free aquifer

In the carbonate-free aquifer, the changes in reduction potentials depend primarily on the stoichiometric coefficients of protons in the reduction reactions (Table 1). For example, the reduction of H^+^ to H_2_ consumes 8 protons per 8 electrons, while the reduction of Fe^2+^ to goethite consumes 24 protons per 8 electrons. As a result, the increase in the reduction potential of the redox couple of H^+^/H_2_ is the smallest, 267.8 mV, while the increase for the couple of Fe^2+^/goethite is the largest, 639.5 mV. For other redox couples, their reduction reactions consume 9–10 protons per 8 electrons, close to the stoichiometric coefficient of protons in the redox couple of H^+^/H_2_. As a result, the changes in the reduction potentials of these redox couples are larger than, but close to, the change in the potential of H^+^ reduction to H_2_.

#### Calcite-rich aquifer

Compared to those in the carbonate-free aquifer, the increases in reduction potentials are relatively small in the calcite-rich aquifer. Where CO_2_ partial pressure increases from near 0 to 30 atm, the reduction potential of H^+^/H_2_ increases by 176.1 mV, and that of Fe^2+^/goethite increases by 391.2 mV. These small increases arise from the limited decrease in pH (Figure 2C).

For the redox couples of acetate, lactate, propionate, and methanol, their reduction reactions consume bicarbonate (Table 1). As a result, their reduction potentials also vary with bicarbonate concentration or activity. Where CO_2_ partial pressure increases from near 0 to 30 atm, the concentration and hence activity of bicarbonate increases by about one order of magnitude (Figure 2D). The stoichiometric coefficient of bicarbonate varies from 1 per 8 electrons in the reduction reaction of bicarbonate to methane to 2 per 8 electrons in the reduction reactions of acetate and lactate. As a result, the increases in the reduction potentials of acetate and lactate with CO_2_ partial pressure are faster than that of ${\text{HCO}}_{3}^{-}$/methane (Figure 5B).

### Available energy

**Figure 6:**
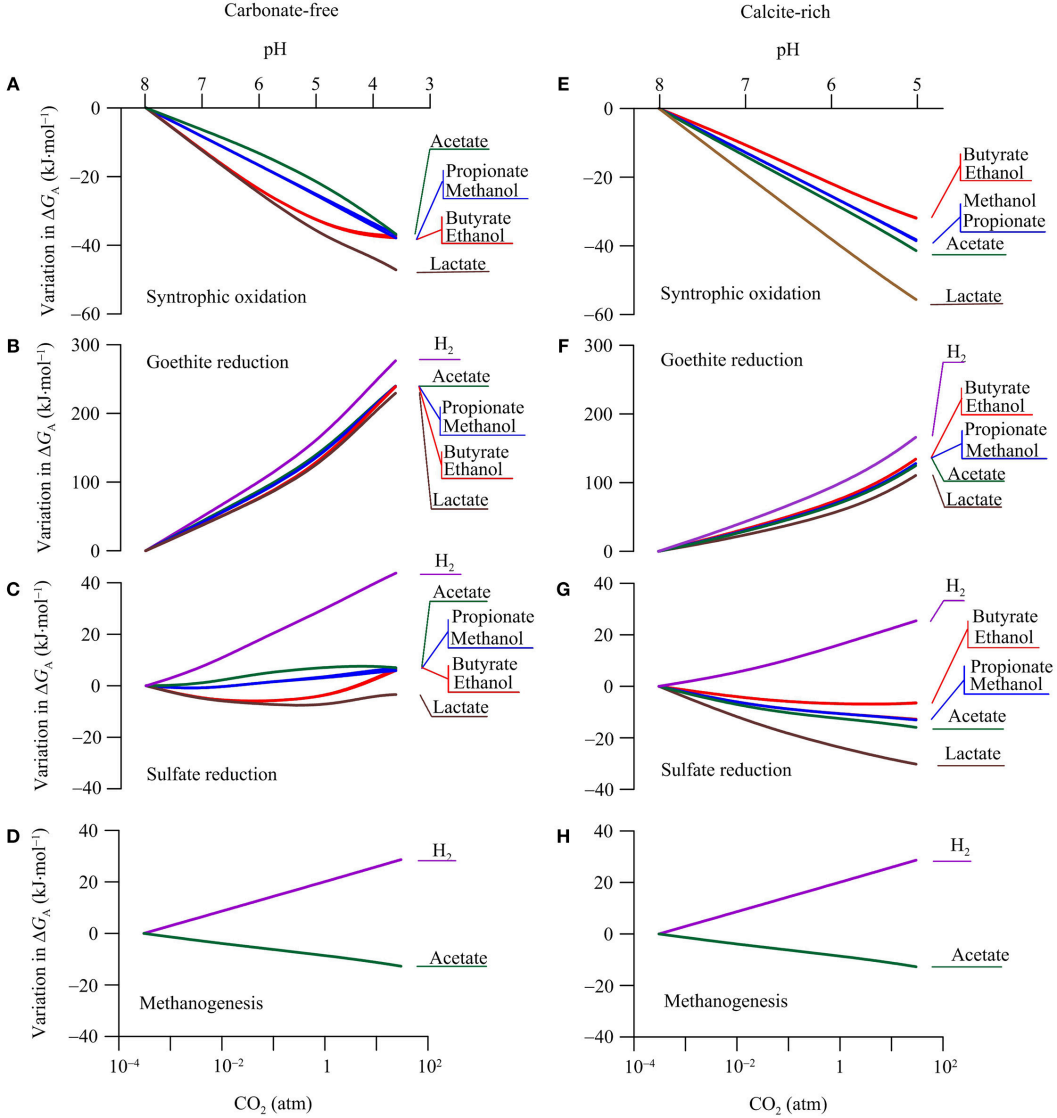
**Variations in the available energy Δ***G***_**A**_ of syntrophic oxidation, goethite reduction, sulfate reduction, and methanogenesis in a hypothetical carbonate-free (A–D)** and calcite-rich **(E–H)** aquifer. Labels show the electron donors of redox reactions; see Table 2 for reaction equations.

The available energy is a key geochemical parameter that controls both the rates of microbial reactions and the growth of functional groups (Jin, 2012). We compute the energy available from the reduction potentials of electron donors and acceptors according to Equation 6. The calculation focuses on the common functional groups in aquifers, including syntrophs, ferric iron reducers, sulfate reducers, and methanogens (Table 2). As in the above case of reduction potentials, our calculation focuses on the variations in the available energy with CO_2_ partial pressure, in order to highlight the responses of microbial reactions. Figure 6 shows how the energy available to the microbial functional groups responds to the increase in CO_2_ partial pressure.

#### Syntrophs

Syntrophs use organic compounds as electron donors and protons as an electron acceptor (Table 2). The above analysis shows that, in the two hypothetical aquifers, CO_2_ addition raises the reduction potentials of organic compounds faster than that of protons (Figure 5). As a result, the increases in CO_2_ partial pressure lower the available energies of syntrophic oxidations (Equation 6, Figures 6A,E).

Variations in the available energy can also be interpreted in terms of reactants and products, including proton, acetate, and bicarbonate. For example, protons are produced in the syntrophic oxidation reactions (Table 2). In both the carbonate-free and calcite-rich aquifers, CO_2_ addition decreases groundwater pH (Figures 2A,C), thereby lowering the available energy. Because of the difference in the pH decrease between the two aquifers, the available energy also decreases to different extents. Taking as examples the syntrophic oxidation of butyrate and ethanol, the available energy decreases more significantly in the carbonate-free aquifer than in the calcite-rich aquifer.

In the carbonate-free aquifer, for both butyrate- and ethanol-oxidizing syntrophs, the variations in the available energy level off at CO_2_ partial pressures greater than 1 atm. This is because the syntrophic oxidation of butyrate and ethanol produces acetate (Table 2). The increase in CO_2_ partial pressure decreases groundwater pH, which in turn decreases the concentration of acetate (Figure 3). At CO_2_ partial pressures above 1 atm, pH decreases below 5, and acetate concentration is less than half of the total concentration of acetate and acetic acid (Figure 3A). The decrease in acetate concentration increases the energy available to butyrate- and ethanol-oxidizing syntrophs, which counteracts the decreases in the available energy by the pH decrease.

On the other hand, in the carbonate-rich aquifer, the available energy of butyrate- and ethanol-oxidizing syntrophs decreases steadily with the increase in CO_2_ partial pressure. This is due to the modest pH decrease in this aquifer. At CO_2_ partial pressure of 1 atm, groundwater pH is about 6, and compared to acetic acid, acetate still remains as the dominant form (Figure 3B).

The syntrophic oxidation of acetate, lactate, propionate, and methanol produces bicarbonate (Table 2). As a result, the energy released by these reactions also depends on the concentrations of bicarbonate; an increase in bicarbonate concentration decreases the available energy. In the calcite-rich aquifer, the decrease in pH is less than that in the carbonate-free aquifer, but the increase in bicarbonate concentration is more significant than in the carbonate-free aquifer (Figure 2). Overall, the effect of increasing bicarbonate concentrations takes its toll, leading to greater decreases in the available energies in the calcite-rich aquifer than in the carbonate-free aquifer.

#### Iron reducers

In both aquifers, the increases in CO_2_ partial pressure raise the reduction potential of ferric iron much faster than those of electron donors (Figure 5). As a result, the energy available to iron reducers increases significantly with the increase in CO_2_ (Figures 6B,F).

Like in the above cases of syntrohic oxidations, pH is also a key parameter in determining the available energy. But in the reduction reactions of goethite (Table 2), protons are the reactants and, as a result, the available energy increases with the decrease in pH. Also because the pH decrease is larger in the carbonate-free aquifer than in the calcite-rich aquifer, the increase in the available energy is more significant in the carbonate-free aquifer than in the calcite-rich aquifer.

In both aquifers, the increase in the available energy varies among different electron donors. Specifically, the increase is most significant for H_2_ oxidation, and least significant for lactate oxidation to acetate. This difference arises from the different stoichiometric coefficients of protons in goethite reduction coupled to the oxidation of different electron donors. The stoichiometric coefficient of protons in H_2_ oxidation is the largest, while that in lactate oxidation to acetate is the smallest (Table 2).

#### Sulfate reducers

For sulfate reducers, changes in energy available in response to CO_2_ addition are mixed (Figures 6C,G). Adding CO_2_ increases the reduction potentials of sulfate and electron donors in both the carbonate-free and calcite-rich aquifers. Because the slopes of increases are similar (Figure 5), the addition of CO_2_ has minimal impact on energy available.

In the calcite-free aquifer (Figure 6C), only the available energy of H_2_-oxidizing sulfate reducers increases notably in response to the CO_2_ increase. Where CO_2_ partial pressure increases from near 0 to 30 atm, the available energy increases by 43.8 kJ·mol^−1^. For sulfate reducers that oxidize other electron donors, their available energy responds to the CO_2_ increase, but only marginally. Specifically, for sulfate reducers that oxidize acetate, propionate, and methanol, increases in available energy are less than 7.0 kJ·mol^−1^. For sulfate reducers that oxidize lactate, butyrate, and ethanol, their available energy first decreases with CO_2_ leakage and then increases, but variations remain less than 7.0 kJ·mol^−1^.

These variations in available energy can be accounted for using reactants and products of sulfate reduction reactions. Specifically, sulfate reduction by the oxidation of H_2_, acetate, propionate, and methanol consumes protons (Table 2). As a result, the available energy increases with the decrease in pH, and the significance of the increase depends on the stoichiometric coefficients of protons. Hydrogenotrophic sulfate reduction consumes most protons, and its available energy increases most significantly with the increase in CO_2_ partial pressure. In comparison, in sulfate reduction by the oxidation of acetate, propionate, and methanol, the stoichiometric coefficients of protons are relatively small, and the increases in available energy in response to decreasing pH are also small.

For acetate-oxidizing sulfate reduction, the increase in the available energy is further limited by the speciation of acetate and acetic acid. As shown in Figure 3A, at CO_2_ partial pressure above 1 atm, increase in the partial pressure decreases significantly acetate concentration, thereby decreasing the available energy.

For sulfate reduction that oxidizes lactate, butyrate, and ethanol, the initial decrease in the available energy can be explained by the production of protons under circumneutral pH condition. In writing the reaction equations for sulfate reduction, we assume that dihydrogen sulfide (H_2_S) is the main species of dissolved sulfide. Under this assumption, no proton is consumed by these reactions (Table 2). But under circumneutral pH conditions, a significant fraction of dissolved sulfide also occurs as monohydrogen sulfide (HS^−^) (Figure 3A). If we replaced H_2_S with HS^−^ in the reaction equations, sulfate reduction by the oxidation of lactate, butyrate, and ethanol would generate protons. This explains the slight decreases in the available energy at the beginning of the CO_2_ increase, where pH of the groundwater is close to 7.

At CO_2_ partial pressure above 0.1 atm, the CO_2_ increase starts to turn groundwater from circumneutral to slightly acidic (pH < 6). Under this condition, H_2_S becomes the only dominant sulfide species, no proton is produced by sulfate reduction, and the available energy is no longer dependent on pH.

At pH below 6, because of the pH control on aqueous speciation (Figure 3A), the decrease in pH also starts to significantly lower acetate concentration. This explains the slight increase in available energy with increasing CO_2_ partial pressure. Note that the speciation effect is relatively small for sulfate reduction by lactate oxidation. This is because lactate oxidation produces acetate, and the concentrations of both acetate and lactate decreases with the increase in CO_2_ partial pressure.

In the calcite-rich aquifer (Figure 6G), the energy available to hydrogenotrophic sulfate reducers increases with the increase in CO_2_ partial pressure. For sulfate reducers using other electron donors, their available energy consistently decreases with the increase of CO_2_. In this aquifer, variations in the available energy result from significant changes in both bicarbonate concentration and pH (Figures 2C,D). Specifically, as discussed for the carbonate-free aquifer, under circumneutral pH condition, sulfate reduction by the oxidation of short-chain fatty acids and primary alcohols generates protons, and thus the available energy decreases with the increase in CO_2_ partial pressure. For sulfate reduction that oxidizes acetate, lactate, propionate, and methanol, the available energy is further decreased by the significant increase in bicarbonate concentrations.

#### Methanogens

The simulation results show that in both the carbonate-free and calcite-rich aquifers, the available energy of hydrogenotrophic methanogenesis increases with the increase of CO_2_ partial pressure, while that of acetoclastic methanogenesis decreases with the CO_2_ increase (Figures 6D,H). The difference between the responses of the two pathways arises from the dependence of the available energy on both pH and the concentrations of acetate and bicarbonate in the groundwater. For hydrogenotrohic methanogenesis, it utilizes protons and bicarbonate as substrates, and hence its available energy increases with the decrease in pH and the increase in bicarbonate concentration. For acetoclastic methanogenesis, its available energy depends on the concentrations of acetate and bicarbonate. In the calcite-rich aquifer, the increase in CO_2_ partial pressure raises significantly bicarbonate concentrations, thereby decreasing the energy available to acetoclastic methanogens. On the other hand, in the carbonate-free aquifer, the significant decrease in pH by the CO_2_ increase converts acetate to acetic acid (Figure 3A), which also decreases the available energy.

### Microbial kinetics

The above simulations demonstrate that the mixing of CO_2_ into groundwater increases or decreases the available energy of different microbial reactions to different extents (Figure 6). The available energy controls metabolic rates of microbial functional groups (Jin and Bethke, 2007). According to the thermodynamically consistent rate law (Equations 7, 10, 13, and 14), increases in the available energy increase non-linearly the rate of microbial respiration and hence the rate of microbial growth. On the other hand, decreases in the available energy decrease the rates of microbial respiration and growth. Therefore, we predict that the addition of CO_2_ into aquifers may accelerate some microbial reactions, but impede the progress of others.

We take as examples microbial sulfate reduction and iron reduction, and apply kinetic modeling to microbial reactions under the influence of CO_2_ addition. Our goal is not to describe CO_2_ leakage from a specific reservoir or into a specific aquifer, but to test the prediction that adding CO_2_ into aquifers modulates the rates of microbial reactions. For this reason, we adopt the following assumptions and simplifications:

First, we simulate geochemical and microbial reactions within a unit volume of the hypothetical calcite-rich aquifer using a flush model (Bethke, 2008). This model is analogous to a chemostat reactor (Tempest, 1970). At each time step of the simulation, a given volume of fresh groundwater is poured into the aquifer, while the same amount of the existing groundwater is removed. Chemostat reactors are often characterized using dilution rate. The simulation assumes that the aquifer has a dilution rate of 10 yr^−1^, which corresponds to a groundwater flow of 1 m·yr^−1^, a typical value for confined aquifers (Chapelle, 2001).

**Figure 7:**
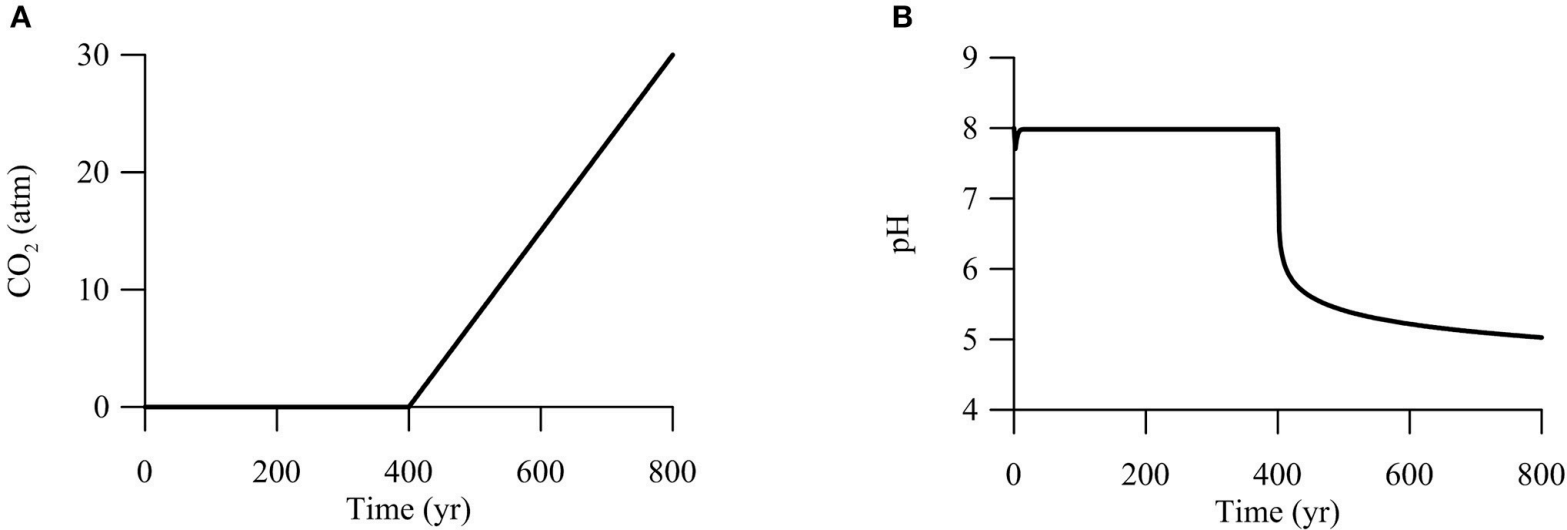
**Variations with time in CO_**2**_ partial pressure (A)** and pH **(B)** in a hypothetical calcite-rich aquifer.

Second, we are interested in CO_2_ leakage during long-term carbon storage. The Intergovernmental Panel on Climate Change recommended that geological carbon storage should store CO_2_ in periods of centuries to millennia (IPCC, 2005). Thus, we set the simulation time at 800 years. The simulation breaks into two phases (Figure 7). During the first 400 years, CO_2_ from deep reservoirs migrates upwards toward the aquifer, but has not reached the aquifer yet. The duration of 400 years is long enough to ensure that microbial metabolisms reach steady state. In the second phase between 400 and 800 years, CO_2_ from the deep reservoirs mixes into the groundwater, raising the partial pressure of CO_2_ linearly to 30 atm at year 800.

Third, simulating microbial reactions requires a series of microbial kinetic, growth, and thermodynamic parameters (Jin and Roden, 2011; Jin et al., 2013). We assume that the values of microbial parameters do not change with environmental conditions, including pH and CO_2_ abundance, and assign the values of microbial parameters on the basis of previous studies (see Table 3). These values have been applied successfully to aquifers (Bethke et al., 2008; Jin and Roden, 2011). The simulation assumes that in the aquifer, H_2_ and acetate, the main products of natural organic matter degradation (McMahon and Chapelle, 1991; Lovley et al., 1994), are produced at the same rate of 1.0 μmol · L^−1^ · yr^−1^. There are few measurements of H_2_ and acetate production in aquifers, and the equal rates are purely assumptive. Nevertheless, the assumed rates are within the ranges reported for aquifers (Chapelle, 2001; Park et al., 2006).

**Table 3:** **Kinetic parameters (rate constant ***k***, and half-saturation constant ***K***_***D***_ and ***K***_***A***_), growth parameters (growth yield ***Y*** and specific maintenance rate ***D***), and thermodynamic parameters (ATP yield ***m***_***P***_ and average stoichiometric number χ) of microbial functional groups**.

**Functional group**	**Redox reaction^a^**	**Kinetic parameter**^**b**^			**Growth parameter**		**Thermodynamic parameter**^**(c)**^	
		***k* (mol·g^−1^·s^−1^)**	***K*_D_ (molal)**	***K*_A_ (molal)**	***Y*^c^ (g · mol^−1^)**	***D*^d^ (s^−1^)**	***m*_P_**	**χ**
Iron reducers	7	1.5 × 10^−5^^e^	1.0 × 10^−6^	7.0^f^	7.8	10^−8^	2.0	8
	8	1.5 × 10^−5^^e^	1.2 × 10^−5^	7.0^f^	5.6	10^−8^	1.5	8
Sulfate reducers	14	1.0 × 10^−6^	1.1 × 10^−6^	3.9 × 10^−5^	5.0	10^−8^	1.0	6
	15	1.0 × 10^−6^	5.0 × 10^−6^	3.9 × 10^−5^	4.6	10^−8^	0.75	6
Methanogens	21	1.0 × 10^−6^	4.7 × 10^−6^	—^g^	1.25	10^−8^	0.25	2
	22	1.0 × 10^−6^	2.3 × 10^−5^	—^g^	2.5	10^−8^	0.5	2

a *See Table 2*.

b *Jin and Roden (2011)*.

c *Jin (2012)*.

d *Price and Sowers (2004)*.

e *Unit is s^−1^*.

f *Unit is g cell dry weight per mol of bioavailable surface sites, i.e., g·mol^−1^*.

g *No electron acceptor dependence*.

The simulation also assumes that the half-saturation constants describe the efficiency of microbes in utilizing the total dissolved electron donors or acceptors, not any specific chemical species. In other words, in computing kinetic factors (Equations 8 and 9), we only account for the total dissolved electron donors and acceptors, or the sum of the concentrations of acids and their conjugate forms.

We carried out the simulation by seeding sulfate and iron reducers with an initial biomass concentration of 1.0 ng · L^−1^. As shown above (Figures 2–4), adding CO_2_ into aquifers can significantly change the chemistry of groundwater. Specifically, at the time of year 400, where CO_2_ starts to mix into the aquifer, groundwater pH decreases immediately from 8 to about 6 (Figure 7B). Afterwards, pH decreases gradually to 5 over the next 400 years. The sharp decrease in pH reflects the fact that a pH drop from 8 to 6 only requires the production of about 1 μM of protons in the groundwater. In the hypothetical calcite-rich aquifer, a relatively small increase in CO_2_ partial pressure from near 0 to 1 atm is sufficient to generate 1 μM of protons (Figure 2C). The subsequent gradual pH decrease can be explained by relatively large change in proton concentrations. An decrease in pH from 6 to 5 requires the production of about 10 μM of protons, which can be generated by raising the partial pressure from 1 to 30 atm and by the simutaneous dissolution of CO_2_ gas and calcite mineral into the groundwater.

#### Sulfate reduction

The above thermodynamic analysis suggests that in the hypothetical calcite-rich aquifer, high CO_2_ may promote hydrogenotrophic sulfate reduction, but inhibit sulfate reducers that oxidize acetate (Figure 6G). We test this prediction by simulating both hydrogenotrophic and acetotrophic sulfate reduction in the aquifer. We assume that sulfate and sulfide in the incoming groundwater have a concentration of 1 mM and 10 μM, respectively. The assumed sulfate concentration is much larger than the half-saturation constant of sulfate reduction (see Table 3), in order to eliminate the limitation of sulfate and to ensure that the two sulfate reducers do not compete with each other for sulfate.

**Figure 8:**
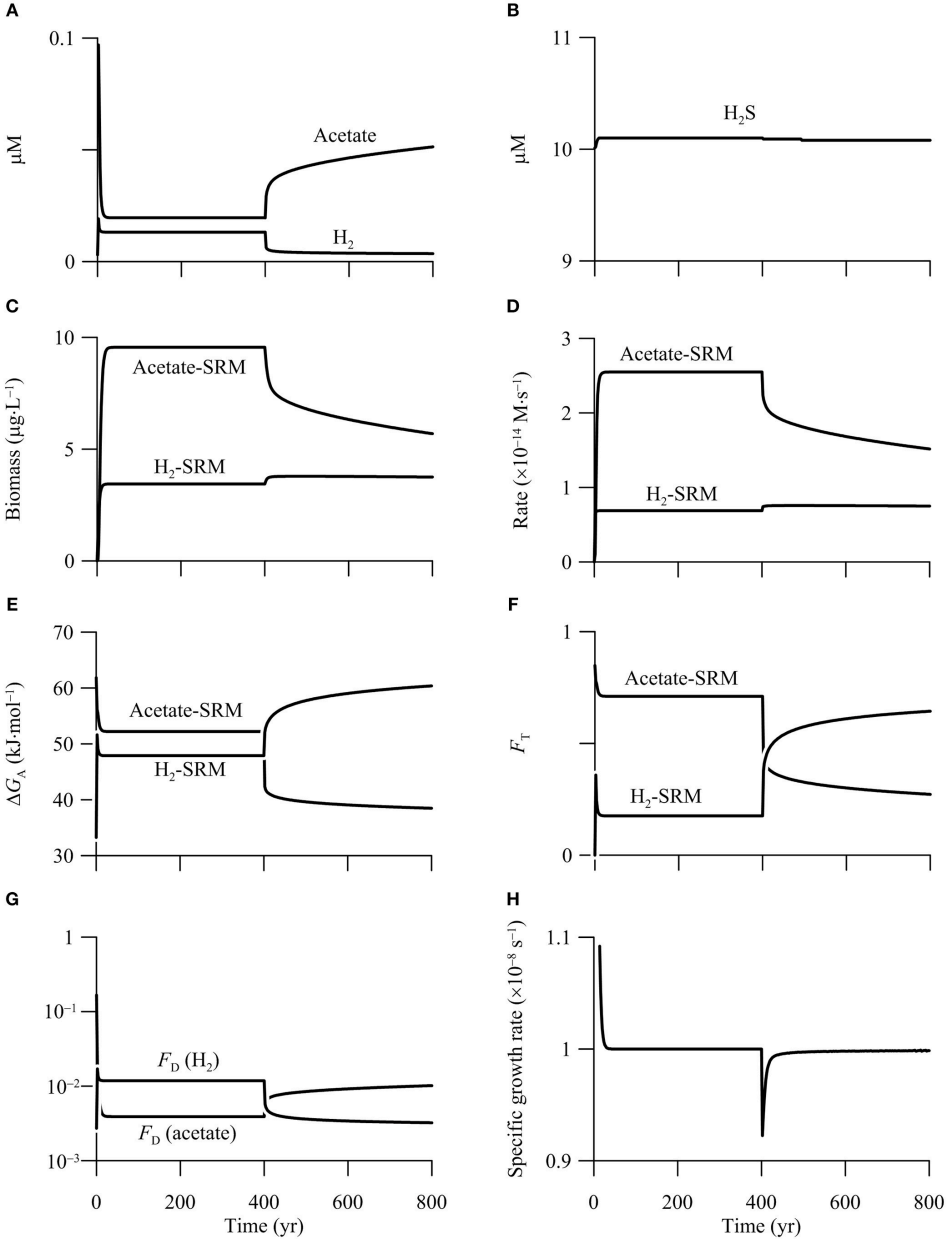
**Variations with time in the concentrations of H_**2**_, acetate (A)**, and sulfide **(B)**, the biomass concentrations of sulfate reducing microbes (SRM) **(C)**, the rates of sulfate reduction **(D)**, the energy available to sulfate reducers **(E)**, the thermodynamic factor *F*_T_ **(F)**, the kinetic factor of H_2_ and acetate *F*_D_ **(G)**, and specific growth rate of acetotrophic sulfate reducer **(H)** in a hypothetical calcite-rich aquifer.

The simulation results suggest that both hydrogenotrophic and acetotrophic sulfate reduction persist in the first simulation phase of 0–400 years (Figure 8), and the hypothetical aquifer can be described as a redox zone of sulfate reduction (Bethke et al., 2011). Specifically, sulfate reduction reaches a steady state after 30 years into the simulation. At steady state, the groundwater contains 13 nM H_2_ and 0.02 mM acetate, the hydrogenotrophic and acetotrophic sulfate reducers grow to a biomass concentration of 3.4 and 9.6 μg · L^−1^, respectively, and the rates of sulfate reduction by oxidizing H_2_ and acetate are 0.2 and 0.8 μmol· L^−1^ · yr^−1^, respectively. Assuming that one cell weighs 10^−12^ g, the hydrogenotroph and acetotroph have a concentration of 3.4 × 10^6^ and 9.6 × 10^6^ cell · L^−1^, respectively.

Although our simulation does not target a specific aquifer, both the predicted cell concentrations and sulfate reduction rates fall within the ranges of confined aquifers. For example, Dockins et al. (1980) applied a synthetic growth medium and the method of Most Probable Number (MPN) to the aquifers of the Northern Powder River Basin, Montana, USA, and reported that sulfate reducers ranged from 20 to >2.4 × 10^5^ cell · L^−1^. Because the MPN method likely underestimated cell concentration by two to three orders of magnitude (Vester and Ingvorsen, 1998), the actual concentrations might have ranged from 1000 to 10^8^ cell · L^−1^.

Previous experimental studies analyzed sulfate reduction rates using ^35^S as a radiotracer. In the same study of Dockins et al. (1980), they determined a sulfate reduction rate of 42.8 μmol · L^−1^ · yr^−1^. Phelps et al. (1994) reported that sulfate reduction rates ranged from <0.1 to 34 μmol · L^−1^ · yr^−1^ in the Atlantic Coastal Plain aquifers. Sulfate reduction rates can also be estimated using geochemical reaction modeling. Thorstenson et al. (1979) estimated a sulfate reduction rate of 0.16 μmol · L^−1^ · yr^−1^ in the Fox Hills-basal Hell Creek Aquifer in North and South Dakota. Murphy and Schramke (1998) estimated that sulfate reduction rate varied from 1.3 × 10^−3^ to 0.25 μmol · L^−1^ · yr^−1^ in the Middendorf aquifer, one of the Atlantic Coastal Plain aquifer system. The consistency between our simulation and previous efforts suggests that despite the simplifications and assumptions, our model can be applied to microbial reactions in aquifers.

During the second phase of 400–800 years, the increase in CO_2_ partial pressure inhibits the metabolism of acetotrophic sulfate reducer, a result that agrees with the prediction based on reaction thermodynamics. Specifically, both the biomass concentration and sulfate reduction rate decrease sharply—by 29%—during the first 90 years. Afterwards, the biomass concentration and rate decrease almost linearly with time to 5.7 μg · L^−1^ and 0.48 μmol · L^−1^ · yr^−1^, respectively, at year 800.

The inhibition comes from the decrease in the available energy, and can be evaluated using the thermodynamic factor *F*_T_. This factor quantifies how the available energy, relative to the saved energy, controls microbial rate. As shown in Figure 8E, before the CO_2_ addition, energy available to acetotrophic sulfate reducers is 52.2 kJ · mol^−1^, larger than the saved energy, which is 33.75 kJ · mol^−1^ (Equation 11 and Table 3). As a result, the thermodynamic factor takes a value of 0.7 (Figure 8F). In the second phase, the available energy drops by 12.5 kJ · mol^−1^ during the first 90 years, and then decreases gradually to 38.5 kJ · mol^−1^ at year 800. The decrease in the available energy pulls down the thermodynamic factor to 0.27 at year 800.

The simulation also predicts that the CO_2_ addition ultimately drives acetotrophic sulfate reducers out of the aquifer. As shown in Figure 8G, in addition to the thermodynamic factor, acetotrophic sulfate reduction is also limited by acetate, the significance of which is quantified using the kinetic factor *F*_D_. Before the CO_2_ addition, because of small acetate concentration, the kinetic factor *F*_D_ is only about 3.9 × 10^−3^. Note that the kinetic factor of sulfate is close to unity, because of the large sulfate concentration. Substituting these values, together with the rate constant and growth yield (see Table 3) to the rate law (Equations 7 and 14), acetotrophic sulfate reducers take a specific growth rate of 1.0 × 10^−8^ s^−1^, which equates the assumed rate of specific maintenance, and allows the growth to reach a steady state.

But after the addition of CO_2_ into the aquifer, the specific growth rate decreases because of the decrease in the available energy and the rate of sulfate reduction. Although the deceleration of acetotrophic sulfate reduction raises the concentration and hence the kinetic factor of acetate (Figures 8A,G), the increase is not sufficient to offset the decrease by decreasing available energy. As a result, the specific growth rate decreases below the specific maintenance rate, and the population starts to decline. Given sufficient time, acetotrophic sulfate reducers will disappear (result not shown).

During the second phase of 400–800 years, the addtion of CO_2_ raises the rate of hydrogenotrophic sulfate reduction, but only to a limited extent. According to the simulation results (Figures 8C,D), the biomass concentration and sulfate reduction rate increase, but only slightly—about 10%. Both the rate and biomass concentration reach maximum values after 20 years into the second phase, and remain nearly constant thereafter.

The modest response arises from the opposing effects of the increasing available energy and the decreasing H_2_ concentration. As shown in Figures 8E,F, before the CO_2_ addition, the energy available to hydrogenotrophic sulfate reducers is 47.9 kJ · mol^−1^, close to the saved energy, which is 45 kJ · mol^−1^ (Equation 11 and Table 3). The thermodynamic factor *F*_T_ takes a value of about 0.2. H_2_ concentration is also smaller than the assumed half-saturation constant of 1.1 μM (Table 3), and the kinetic factor *F*_D_ takes a value of 0.012.

After the addition of CO_2_, the available energy increases, raising the thermodynamic factor *F*_T_. At year 800, available energy increases to 60.4 kJ · mol^−1^, and the thermodynamic factor increases to 0.64. On the other hand, because the rate of H_2_ production by organic mater degradation is held constant, the increase in the H_2_ oxidation rate decreases the concentration of H_2_, decreasing the kinetic factor *F*_D_. At year 800, H_2_ concentration decreases to 3.6 nM, and the kinetic factor *F*_D_ decreases to 0.003. Because the increase in rate by the increasing available energy nearly balances the decrease by decreasing H_2_ concentration, the sulfate reduction rate does not vary significantly.

#### Microbial competition

In aquifers, microbial sulfate reduction and iron reduction may take place at the same location, competing against each other for electron donors (Flynn et al., 2013; Maamar et al., 2015). The above thermodynamic analysis suggests that in the hypothetical calcite-rich aquifer, high CO_2_ promotes microbial iron reduction, but inhibits sulfate reducers that utilize short-chain fatty acids (Figures 6F,G). As a result, the addition of CO_2_ into aquifers may change the outcome of the competition between iron reducers and sulfate reducers.

To test this prediction, we simulate the metabolisms of iron reducers and sulfate reducers that oxidize H_2_ and acetate in the hypothetical calcite-rich aquifer. We assume that the aquifer has a porosity of 0.15 and contains 1% goethite, and that sulfate, sulfide, and ferrous iron in the incoming groundwater has a concentration of 1.0 mM, 10 μM, and 10 μM, respectively. The simulation also includes the metabolisms of hydrogenotrophic and acetoclastic methanogens. In this way, a total of six functional groups are considered in the simulation.

**Figure 9:**
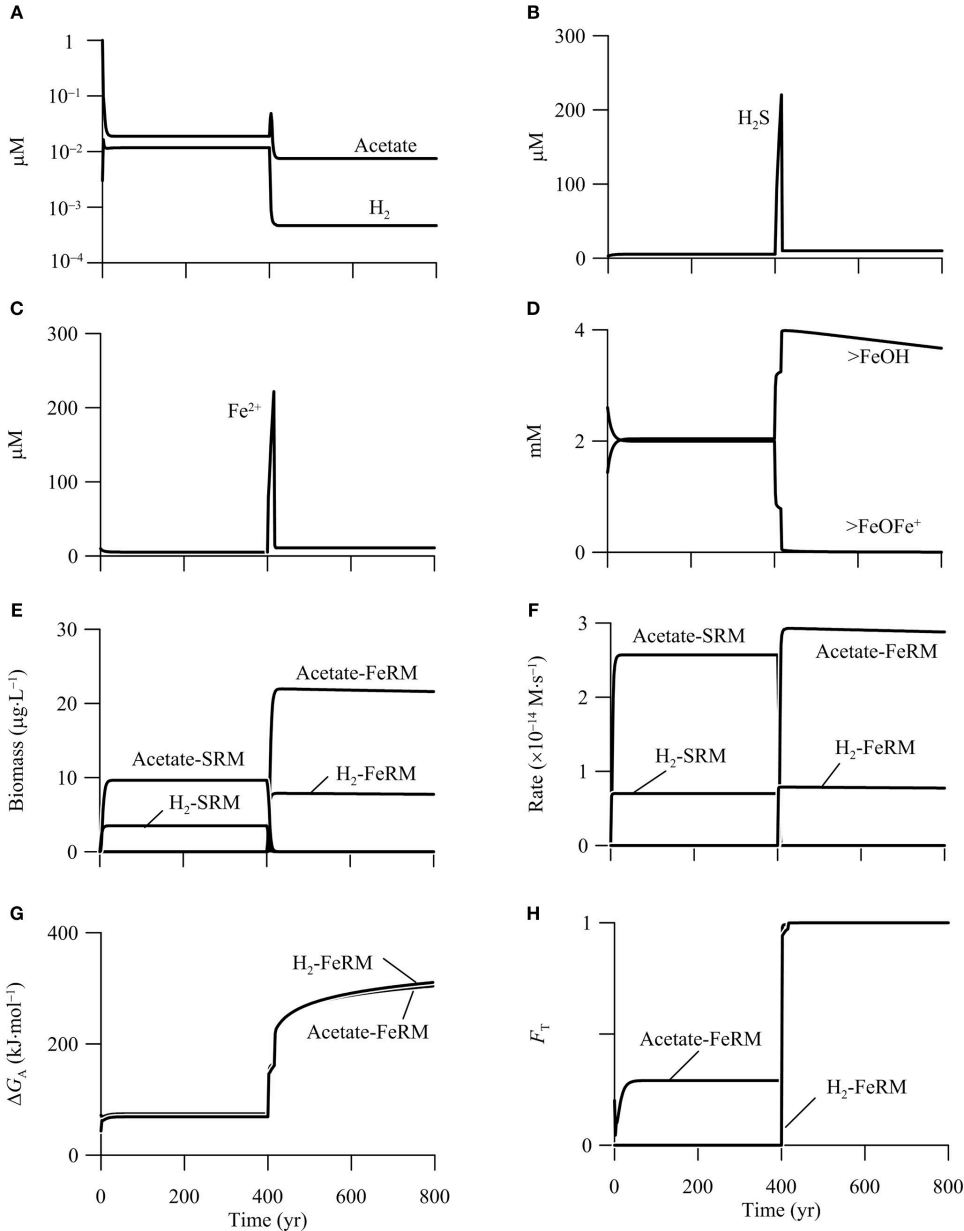
**Variations with time in the concentrations of H_**2**_, acetate, (A)**, sulfide **(B)**, ferrous iron **(C)**, the bioavailable surface sites >FeOH, and sorbed ferrous iron >FeOFe^+^ **(D)**, the biomass concentrations of iron reducing (FeRM) and sulfate reducing microbes (SRM) **(E)**, the rates of iron reduction and sulfate reduction **(F)**, the energy available to iron reducers **(G)**, and the thermodynamic factor *F*_T_ of iron reducers **(H)** in a hypothetical calcite-rich aquifer.

Figure 9 shows the results of the simulation. In the first 400 years, before the addition of CO_2_, out of the six functional groups, only the two sulfate reducers survive in the aquifer. The simulated rates and biomass concentrations are the same as those in the first phase of the above example (Figure 8). In addition, there are two chemical species on the surface of goethite, free or bioavailable surface sites (>FeOH) and sorbed ferrous iron (>FeOFe^+^), and their bulk concentrations are about 2 mM (Figure 9D).

Between year 400 and 800, the CO_2_ addition promotes the metabolism of acetotrophic and hydrogenotrophic iron reducers, and excludes the two sulfate reducers from the aquifer. In other words, the CO_2_ addition changes the aquifer from a sulfate-reduction zone to a zone of iron reduction. At steady state, hydrogenotrophic and acetotrophic iron reducers reach a concentration of 7.8 and 21.9 μg · L^−1^, respectively. Hydrogenotrophic and acetotrophic iron reduction proceeds at a rate of 0.2 and 0.9 μmol · L^−1^ · yr^−1^, respectively (Figures 9E,F).

The CO_2_ addition promotes iron reduction by raising the energy available from the reduction of goethite. As shown in Figure 9G, the CO_2_ addition raises significantly the energy available to iron reducers. At year 400, the available energy is only 69.0 kJ · mol^−1^ for hydrogenotrophic iron reduction, less than the saved energy of 90.0 kJ · mol^−1^ (Equation 11 and Table 3). The available energy is 74.3 kJ · mol^−1^ for acetotrophic iron reduction, slightly larger than the saved energy of 67.5 kJ · mol^−1^. Within 90 years, because of the sharp decrease in pH, the available energy of both hydrogenotrophic and acetotrophic iron reduction increases to about 270 kJ · mol^−1^. As a result, the thermodynamic factors of the two iron reducers increase to near unity at year 490, and stay close to unity afterwards (Figure 9H).

CO_2_ addition also promotes microbial iron reduction by increasing the concentration of bioavailable surface sites (Figure 9D). According to the rate law (Equation 12), the rate of microbial iron reduction depends on the concentration of bioavailable surface sites of ferric minerals, which in turn depends on the sorption of ferrous iron. Ferrous iron sorption is controlled by pH; more ferrous iron sorbs onto the surface sites of goethite at high pH, and vice versa (Dixit and Hering, 2006). As shown in Figure 9D, the decrease in pH removes the sorbed ferrous iron from the surface sites, and thus makes available nearly all of the surface sites to bioreduction.

The increases in the available energy and the concentration of bioavailable surface sites raise the rates of iron reduction, which enable both hydrogenotrophic and acetotrophic iron reducers to compete successfully against sulfate reducers. Specifically, acetate and H_2_ oxidation by the iron reducers decrease acetate and H_2_ concentrations below 7.5 and 0.5 nM, respectively (Figure 9A). The small acetate and H_2_ concentrations decreases the specific growth rates of sulfate reducers below specific maintenance rates, which leads to the death of the sulfate reducers (Bethke et al., 2008).

Hydrogenotrophic and acetoclastic methanogens do not survive in the hypothetical aquifer, either before or after the addition of CO_2_. The absence of methanogens is accounted for by the limited availability of electron donors, and by the relatively small yields *Y* of biomass synthesis. For example, because of the small acetate concentration, the kinetic factor *F*_D_ of acetate for acetoclastic methanogen is very small, only 8.2 × 10^−4^. Neglecting the thermodynamic control, and substituting the kinetic factor and the growth yield to the rate law (Equations 7 and 13, Table 3), the methanogen has a specific growth rate of 2.1 × 10^−9^ s^−1^, smaller than the assumed specific maintenance rate of 10^−8^ s^−1^.

Note that in the above two examples of kinetic simulation, we may have underestimated microbial responses to CO_2_ addition. Specifically, because microbial parameters are assumed constant, the predicted microbial responses arise mostly from the thermodynamic impact of CO_2_ addition. However, microbial parameters may not be constant, but depend on the physicochemical conditions, including the pH, temperature, pressure, and salinity of the environment (Ingraham, 1987; Jin et al., 2013). In the second phase of the simulations, CO_2_ partial pressure is assumed to increase linearly with time, giving rise to a continuous decrease in groundwater pH (Figure 7). We speculate that, because of the impact of CO_2_ and pH on microbial physiology, actual microbial responses would be more complex than what we have demonstrated here.

### Discussion

The above modeling exercises analyzed how the thermodynamics and kinetics of microbial reactions respond to the addition of CO_2_ into aquifers. The simulation is based on a simple mixing model that adds CO_2_ gas into two different hypothetical aquifers—a carbonate-free aquifer of limited pH buffering capacity and a calcite-rich aquifer that effectively buffers the change of pH (Figure 1). The addition of CO_2_ gas would occur where CO_2_ from deep reservoirs migrates into the aquifers in the phase of gas.

The simulation focused on syntrophic oxidation, iron reduction, sulfate reduction, and methanogenesis (Tables 1, 2). According to the simulation results, high CO_2_ raises the reduction potentials of electron donors and acceptors in the two hypothetical aquifers. The increases are different for different electron donors and acceptors and ultimately determine how, and to what extent, energy available to different microbes changes.

Considering the control of available energy on microbial kinetics (Jin et al., 2013), rates of different microbial reactions may respond to the addition of CO_2_ in different ways and to different degrees. These predictions are supported by the results of two kinetic simulations of microbial reactions in the hypothetical calcite-rich aquifer. The different responses of individual microbial reactions not only control the outcome of microbial interaction, but also bear on the effort of geological carbon sequestration.

#### Syntrophic oxidation

The simulation results show that in the two hypothetical aquifers, the increases in CO_2_ partial pressure lower the available energy of syntrophic oxidation (Figures 6A,E). Based on the relationship between thermodynamics and kinetics (Equation 7), we predict that increases in CO_2_ partial pressure may impede the progress of syntrophic oxidation of organic compounds.

Previous experimental studies investigated the impact of CO_2_ on acetate consumption by both model syntrophs and natural consortia. For example, Kato et al. (2014) analyzed acetate consumption by *Thermacetogenium phaeum*, a representative microbe capable of syntrophic oxidation of acetate to bicarbonate and dihydrogen (reaction 1 in Table 2). They also included in their experiments *Methanothermobacter thermautotrophicus*. *M. thermautotrophicus* makes methane by scavenging H_2_, maintaining H_2_ partial pressure at low levels, <3.0 × 10^−4^ atm (reaction 21) (Kato et al., 2014, their Figure 1C). Kato et al. (2014) grew the two microbes in batch reactors at 55°C, and monitored the accumulation of methane under different CO_2_ partial pressures. We calculated acetate consumption rates from the reported temporal variations of methane concentrations (Kato et al., 2014, their Figure 4B). These rates show that the increases in CO_2_ partial pressure slowed down the consumption of acetate. Specifically, the rate was about 3.3 mM·d^−1^ at the partial pressure near 0 atm, 1.8 mM·d^−1^ at the partial pressure of 0.2 atm, and only 0.3 mM·d^−1^ at the partial pressure of 1.0 atm.

Mayumi et al. (2013) analyzed acetate consumption by natural consortia from an oil reservoir. They grew the consortia at 55°C and different CO_2_ partial pressures, and analyzed the occurrence of syntrophic acetate oxidation. According to their results, at the CO_2_ partial pressure of 0.04 atm, syntrophic oxidation dominated the consumption of acetate. But at the partial pressure of 2.0 atm, syntrophic oxidation became thermodynamically unfavorable. The results of these experimental studies agree with the inhibitory effect of CO_2_ on syntrophic oxidations.

#### Microbial iron and sulfate reduction

According to the simulation results, the thermodynamics of microbial iron reduction and sulfate reduction respond differently to the changes in CO_2_ partial pressure. The increases in CO_2_ raise significantly the energy available to iron reducers, but change, to a limited extent, the energy available to sulfate reducers (Figures 6B,F). These results compare well with the observations of previous aquifer experiments.

Kharaka et al. (2006) injected about 1600 t of CO_2_ into a regional aquifer of 1500 m depth in the US Gulf Coast. Kharaka et al. (2010) also injected 9 t of CO_2_ into a shallow gravel aquifer in Montana, USA. Kirk (2011) computed the variations in the energy available to iron reducers during the two experiments. In the regional aquifer, the CO_2_ injection increased the energy available to acetotrophic and hydrogenotrophic iron reducers by about 47 and 62 kJ·mol^−1^, respectively. In the shallow aquifer, the energy available to acetotrophic and hydrogenotrophic iron reducers increased by 77 kJ· and 88 kJ·mol^−1^, respectively.

It is appropriate to compare the observations of the two field experiments to the simulation results of the calcite-rich aquifer. First, calcite is present in the test layer of the regional aquifer, a quartz and feldspar sandstone (Kharaka et al., 2006). Limestone occurs at notable fractions in the shallow aquifer (Kharaka et al., 2010). Second, during both aquifer experiments, in addition to the pH decreases, the CO_2_ injections increased significantly the concentrations of bicarbonate and calcium (Kharaka et al., 2006, 2009, 2010). These chemical variations support the occurrence of carbonate mineral buffering during the CO_2_ injections.

In both field experiments, the thermodynamic responses of microbial iron reduction are consistent with our simulation results—the increases in CO_2_ levels raised the energy available to iron reducers, and the increase was larger for hydrogenotrophs than for acetotrophs. But the sizes of the increases were only about half of our modeling results. In the hypothetical calcite-rich aquifer, the increases in CO_2_ partial pressure raise the energy available to acetotrophic and hydrogenotrophic iron reducers by 124 and 166 kJ·mol^−1^, respectively (Figure 6F).

These differences arise from the difference in the magnitude of pH decrease between our simulations and the field experiments. Specifically, in our model, the pH of the groundwater is assumed at 8.0, and it drops to 5.0 in response to CO_2_ leakage. In comparison, the pH decreased from 7.2 before the injection to 5.7 after the injection in the regional aquifer of the US Gulf Coast, and from 7.0 to 5.6 in the shallow aquifer in Montana, USA (Kharaka et al., 2009, 2010).

If we apply the pH variations of the field experiments to the hypothetical calcite-rich aquifer model, we would arrive at thermodynamic responses similar to those in the field experiments. Taking an initial pH of 7.0 for the groundwater, and by raising CO_2_ partial pressure to 5.5 atm, the pH would drop to 5.5, and the energy available to acetotrophic and hydrogenotrophic iron reducers would increase by 71.4 and 91.3 kJ·mol^−1^, respectively, close to the changes in the field experiments (Kirk, 2011).

According to Kirk (2011), during the field experiments, energy available to microbial sulfate reduction varied little. Specifically, for acetotrophic sulfate reduction (reaction 15 in Table 2), the CO_2_ injection decreased the available energy by 3 kJ·mol^−1^ in the regional aquifer, and increased by 5 kJ·mol^−1^ in the shallow aquifer. For hydrogenotrophic sulfate reduction (reaction 14), the CO_2_ injection increased the available energy by about 12 kJ·mol^−1^ in both aquifers. These variations are similar in magnitude as the results of our simulation (Figure 6G).

#### Methanogenesis

The simulation results show that increases in CO_2_ partial pressure raise the energy available to hydrogenotrophic methanogenesis, but lower the available energy of acetoclastic methanogens. These results suggest that high CO_2_ may promote hydrogenotrophic methanogensis, but inhibit the metabolism of acetoclastic methanogens.

The predicted benefit to hydrogenotrophic methanogenesis is supported by the field experiments of O'mullan et al. (2015). O'mullan et al. (2015) carried out two push-pull tests in a shallow aquifer in the Newark Basin, USA. During the tests, they first injected CO_2_-enriched solution into the aquifer through a test well, and then extracted the groundwater from the same well. They performed geochemical and microbiological characterizations of groundwater before and after the CO_2_ injections. According to their results, the CO_2_ injection triggered significant changes in microbial community composition, including an increase in the relative abundance of 16S rRNA gene sequences that belong to *Methanomicrobia* and *Methanobacteria*.

Members of *Methanobacteria* can make methane using H_2_, but not acetate (Whitman et al., 2006). Thus, the increase in their gene sequences confirms the modeling prediction that high CO_2_ stimualtes the metabolism of hydrogenotrophic methanogens. On the other hand, *Methanomicrobia* includes members capable of both hydrogenotrophic and acetoclastic methanogenesis. It is unclear whether or not the increases in the sequences of *Methanomicrobia* also arose from the response of hydrogenotrophic methanogenesis.

The predicted inhibition of acetotrphic methanogenesis is supported by the laboratory experiments of Kato et al. (2014). They examined the metabolism of *Methanosarcina thermophila* in batch reactors under different CO_2_ partial pressures. In their experiments, *M. thermophila* grew by converting acetate to methane, and its growth rate decreased with increases in CO_2_ partial pressure. Specifically, the rate at 1.0 atm CO_2_ was about 89% of the rate at partial pressure near 0 atm (Kato et al., 2014, their Figures 4A,C).

#### Microbial interaction

Microbial reactions rarely occur in isolation in aquifers, but mingled with each other, forming complex networks of metabolic interactions. By acting on individual microbial reactions, CO_2_ plays a role in the outcome of microbial interactions.

We illustrated the role of CO_2_ in microbial interactions by carrying out two kinetic simulations of microbial metabolisms in the hypothetical calcite-rich aquifer. The first simulation focused on the responses of H_2_- and acetate-oxidizing sulfate reducers, and the second explored the competition between sulfate reducers and iron reducers. The results showed that CO_2_ controls the outcome of microbial interaction, and that high CO_2_ favors microbial iron reduction over sulfate reduction. These results are supported by the observations of previous laboratory and field experiments.

Kirk et al. (2013) studied how CO_2_ abundance affected the competition between natural communities of iron and sulfate reducers. They grew microbial consortia from a freshwater aquifer in fed-batch reactors using acetate-based media under different CO_2_ partial pressures. According to their observations, at CO_2_ partial pressure of 0.02 atm, the reactors had a pH of 7.2, and both iron and sulfate reduction took place. But sulfate reduction overwhelmed iron reduction; sulfate reducers consumed 85% of acetate, and iron reducers used the remaining 15%. In contrast, in reactors with 1.0 atm CO_2_ partial pressure, the pH dropped to 5.7, and iron reducers won the competition against sulfate reducers. Iron reduction consumed at least 90% of acetate while sulfate reduction consumed a negligible portion (<1%).

The control of CO_2_ partial pressure is further supported by the difference in microbial community composition (Kirk et al., 2013). 16S rRNA gene analysis shows that sequences grouped within *Geobacteraceae* and *Myxococcaceae* were more than twice as abundant in the reactors with 1.0 atm CO_2_ as in those with 0.02 atm CO_2_. Members of *Geobacteraceae* and *Myxococcaceae*, such as *Geobacter* and *Anaeromyxobacter*, are capble of iron reduction (Lonergan et al., 1996; Treude et al., 2003).

The aforementioned field experiments of O'mullan et al. (2015) also support the role of CO_2_ in microbial competition. Specifically, in their two push-pull tests, the CO_2_ injection increased the relative abundance of 16S rRNA genes associated with iron reducers, including *Geothrix* and *Geobacter*. The increase in these genes overlapped in time with the increase in the concentration of groundwater ferrous iron (O'mullan et al., 2015, their Figures 1, 7). These observations suggest that the CO_2_ injection may have stimulated microbial reduction of ferric minerals.

O'mullan et al. (2015) also detected an increase of genes associated with *Desulfosporosinus* in the second push-pull test. Members of *Desulfosporosinus* can use sulfate as an electron acceptor, but some members are also capable of reducing ferric minerals under acidic conditions (Senko et al., 2009; Bertel et al., 2012). We note that during the test, the increase in the genes of *Desulfosporosinus* occurred after the decrease of groundwater pH, and coincided with the increase in the genes of *Geothrix* and *Geobacter*, and with the increase in the concentrations of ferrous iron and sulfate (O'mullan et al., 2015, their Figures 1, 7). We suspect that the increase of *Desulfosporosinus*-associated genes likely reflects an increase in microbial iron reduction, not sulfate reduction.

#### Microbial community

The two kinetic simulations underscore the importance of microbial community membership in applying the results of biogeochemical modeling. Specifically, the impact of CO_2_ addition on sulfate reducers depends on the presence of iron reducers. The first kinetic simulation represents a case in which aquifers house only sulfate reducers, but not iron reducers. The results show that CO_2_ addition promotes, to a limited extent, hydrogenotrophic reduction of sulfate, and limits significantly the progress of acetotrophic sulfate reduction (Figure 8).

The second kinetic simulation represents a case where both iron reducers and sulfate reducers are present. According to the simulation results (Figure 9), the presence of iron reducers dramatically changes the fate of sulfate reducers during the addition of CO_2_. Before CO_2_ is added into the aquifer, acetotrophic and hydrogenotrophic sulfate reducers win the competition against iron reducers and thrive in the aquifer. But the CO_2_ addition wipes the sulfate reducers out, and allows iron reducers to flourish in the aquifer.

The importance of community membership resonates with previous investigations on the response of acetocalstic methanogens to CO_2_. As discussed above, our modeling results predict that high CO_2_ inhibits acetoclastic methanogenesis. This prediction is supported by the experiments of Kato et al. (2014) on the pure culture of *M. thermophila*. But the inhibitory effect can be reversed where methanogens live together with syntrophs.

For example, in the same study, Kato et al. (2014) also analyzed acetate consumption by *M. thermophila* in the presence of *T. phaeum* and *M. thermautotrophicus*. In these experiments, *T. phaeum* and *M. thermautotrophicus* carried out acetate syntrophic oxidation and hydrogenotrophic methanogenesis to make methane, competing against *M. thermophila* for acetate. According to the experimental observations (Kato et al., 2014, their Figures 1, 2), high CO_2_ did not inhibit, but promoted, the significance of acetoclastic methanogenesis by *M. thermophila*.

The above-mentioned experiments of Mayumi et al. (2013) also contradict the inhibitory effect of high CO_2_ on acetoclastic methanogenesis. In their microcosm experiments, both syntrophs and methanogens were capable of using acetate. At 0.04 atm of CO_2_, syntrophic oxidation dominated the consumption of acetate, but acetoclastic methanogenesis took over at 2.0 atm CO_2_.

The contradiction between the model predictions and the experimental observations arises from the competition between syntrophs and methanogens. In these experiments, syntrophs won the competition against acetoclastic methanogens at low CO_2_. Increases in CO_2_ lowered the energy available to syntrophs, limiting the progress of syntrophic oxidation. High CO_2_ also decreased the energy available to acetoclastic methanogens, but compared to that of syntrophic oxidation, the decrease was modest (Figures 6A,D,E,H; Mayumi et al., 2013, their Figure 4; Kato et al., 2014, their Figure 6). As a result, high CO_2_ helped acetocalstic methanogens win the competion against syntrophs, which led to the apparent promotion of acetoclastic methanogenesis. In applying the modeling results of methanogenesis to natural environments, we should consider how methanogens interact with other microbial reactions.

#### CO_2_ trapping

Many microbial reactions consume protons, and thus have the potential of trapping CO_2_ (Table 2). By consuming protons, these reactions drive forward CO_2_ hydrolysis (Equation 1), converting CO_2_ into bicarbonate. In this way, carbon can be more securely stored within the aqueous phase and potentially precipitate as carbonate minerals, such as calcite, magnesite (MgCO_3_), and siderite (FeCO_3_). Mineral trapping is considered to be the most secure form of subsurface carbon trapping (Gunter et al., 1997).

The predicted changes in microbial activity by CO_2_ addition are favorable for CO_2_ trapping. Because iron reduction consumes many more protons than sulfate reduction or methanogenesis (Table 2), it has a much greater potential to generate bicarbonate. Per mole of acetate consumed, for example, iron reduction can generate 17 moles of bicarbonate whereas sulfate reduction only generates 3 moles of bicarbonate. As CO_2_ is added into aquifers, a shift toward iron reduction would increase conversion of CO_2_ into bicarbonate. Thus, an increase in the rate of iron reduction relative to the other reactions would act as a positive feedback mechanism on CO_2_ trapping (Kirk et al., 2013).

Although it is well established that microbial reactions help neutralize acid mine water (e.g., Tuttle et al., 1969; Dean et al., 2013; Lindsay et al., 2015), the possibility that they could provide the same ecosystem service in geological carbon storage settings has received relatively little attention. In carbonate aquifers, reaction between carbonic acid and carbonate minerals is likely the dominant source of bicarbonate production. However, in silicic aquifers, we hypothesize that the bicarbonate contribution of microbial reactions can be dominant, depending on the rate at which electron donors are supplied. Where the flux of electron donors into the system is relatively high, microbial reactions have the potential to generate bicarbonate more rapidly than mineral reactions, and may become a major force of CO_2_ trapping. Predicting the fate of CO_2_ within such systems should account for microbial reactions (Kirk et al., 2013).

#### Closing comments

We carried out biogeochemical modeling to analyze how CO_2_ addition impacts the thermodynamics and kinetics of microbial reactions in aquifers. Our simulation is limited in the description of CO_2_ addition and microbial reactions. First, the simulation is based on a simple model of groundwater and CO_2_ mixing. As a result, the application of our modeling results is limited to the addition of CO_2_ gas into aquifers.

However, CO_2_ leakage may be accompanied by reservoir fluids, which are briny and rich in hydrocarbons and gases. The leakage of reservoir fluids into aquifers changes the pH, ionic strength, electron donor and acceptor concentrations, and other chemical properties of groundwater, which in turn influence the thermodynamics of microbial reactions. The reservoir fluids also affect the viability and metabolism of aquifer microbes, which determine how microbes respond to the addition of CO_2_. In these cases, the simulation needs to account for the chemical compositions of reservoir fluids, and a reactive transport modeling can be applied to consider how the rates of reservoir leakage and groundwater flow determine the thermodynamics and kinetics of microbial reactions (Carroll et al., 2009).

Second, the simulation focused on the overall reactions of microbial respiration, and described microbial kinetics using the Monod-type rate laws (Equations 7–14). This approach has been applied successfully to aquifers and other natural environments (Bethke et al., 2008; Jin and Roden, 2011). But the kinetic model only accounts for the concentrations of electron donors and acceptors and the available energy in the environment, without the consideration of microbial physiology or how microbial physiology responds to the changes in the pH of the environment. For example, in response to acidification, cells may decrease their cytoplasmic pH and adjust their pathways, which in turn affect not only biological energy conservation but also microbial growth and maintenance (van Bodegom, 2007; Slonczewski et al., 2009).

Despite the limitations in our model, the simulation results are still illuminating. The results illustrate the complexity in microbiological response to high CO_2_, and are consistent with the observations of previous laboratory and field investigations. Specifically, CO_2_ addition lowers the energy available to syntrophs and to acetoclastic methanogens, but raises the energy available to iron reducers and hydrogenotrophic methanogens. As a result, high CO_2_ may inhibit the metabolisms of syntrophs and acetoclastic methanogens, but promote the metabolisms of iron reducers and hydrogenotrophic methanogens. The simulation results also demonstrate the importance of microbial community membership in applying the simulation results, and show that CO_2_ levels influence the outcome of microbial interactions. Based on these results, we suggest that evaluating the environment impact of geological carbon sequestration should consider the responses of microbial communities.

Our modeling exercises also illustrate the power of coupled thermodynamic and kinetic analysis of microbial reactions. Thermodynamic and kinetic analyses are routine tasks in today's biogeochemical studies. The thermodynamic analysis is on the basis of chemical thermodynamic properties, and tells whether or not, under given geochemical conditions, a microbial reaction is favored by thermodynamics. The kinetic modeling combines thermodynamic properties of chemical substances with kinetic parameters of microbial metabolisms, and predicts how fast microbes catalyze chemical reactions and reproduce themselves.

So far the thermodynamic and kinetic analyses have often been carried out separately. This study combined the two analyses to predict microbial responses to CO_2_ addition. The results of both methods support the conclusion that different microbial reactions respond differently to high CO_2_. Importantly, the kinetic analysis places a quantitative constraint on the thermodynamic predictions. For example, the thermodynamic analysis suggested that CO_2_ addition promotes hydrogenotrophic sulfate reduction. But the kinetic analysis showed that high CO_2_ does not raise significantly the rates of hydrogenotrophic sulfate reduction. The simulation demonstrates a trade-off between the increasing available energy and the decreasing H_2_ availability in the environment where H_2_ production rate remains constant. These simulation results represent example hypotheses generated by the coupled thermodynamic and kinetic modeling that can be further tested using laboratory and field experiments.

## Author contributions

QJ and MK designed the project, and wrote the manuscript; QJ carried out the modeling, and QJ and MK analyzed the results.

## Funding

This research was funded by the National Science Foundation under Award EAR-1636815 and EPS-0903806, and by National Aeronautics and Space Administration under Grant NNX16AJ59G. Publication of this article was funded in part by the Kansas State University Open Access Publishing Fund.

### Conflict of interest statement

The authors declare that the research was conducted in the absence of any commercial or financial relationships that could be construed as a potential conflict of interest.
